# A comprehensive survey of developmental programs reveals a dearth of tree-like lineage graphs and ubiquitous regeneration

**DOI:** 10.1186/s12915-021-01013-4

**Published:** 2021-05-21

**Authors:** Somya Mani, Tsvi Tlusty

**Affiliations:** 1 Center for Soft and Living Matter, Institute for Basic Science (IBS), Ulsan, 44919 South Korea; 2 Department of Physics, Ulsan National Institute of Science and Technology (UNIST), Ulsan, 44919 South Korea

**Keywords:** Development, Asymmetric cell division, Signaling, Homeostatic organism, Cell type lineage graph, Pluripotent, Regeneration

## Abstract

**Background:**

Multicellular organisms are characterized by a wide diversity of forms and complexity despite a restricted set of key molecules and mechanisms at the base of organismal development. Development combines three basic processes—asymmetric cell division, signaling, and gene regulation—in a multitude of ways to create this overwhelming diversity of multicellular life forms. Here, we use a generative model to test the limits to which such processes can be combined to generate multiple differentiation paths during development, and attempt to chart the diversity of multicellular organisms generated.

**Results:**

We sample millions of biologically feasible developmental schemes, allowing us to comment on the statistical properties of cell differentiation trajectories they produce. We characterize model-generated “organisms” using the graph topology of their cell type lineage maps. Remarkably, tree-type lineage differentiation maps are the rarest in our data. Additionally, a majority of the “organisms” generated by our model appear to be endowed with the ability to regenerate using pluripotent cells.

**Conclusions:**

Our results indicate that, in contrast to common views, cell type lineage graphs are unlikely to be tree-like. Instead, they are more likely to be directed acyclic graphs, with multiple lineages converging on the same terminal cell type. Furthermore, the high incidence of pluripotent cells in model-generated organisms stands in line with the long-standing hypothesis that whole body regeneration is an epiphenomenon of development. We discuss experimentally testable predictions of our model and some ways to adapt the generative framework to test additional hypotheses about general features of development.

**Supplementary Information:**

The online version contains supplementary material available at (10.1186/s12915-021-01013-4).

## Background

Contrary to intuition, the key molecules and mechanisms that go into the development of a human (>200 cell types [1]) are the same as those required to produce a hydra (just 7 cell types [2]). More generally, there is a huge diversity of forms and complexity across multicellular organisms, but key molecules of development in *Metazoa* and in multicellular plants are conserved across the respective lineages [3]. The basis of this diversity is illustrated by mathematical models of development which explore possible mechanisms of producing distinctive patterns found in different organisms, for example, segments in *Drosophila* [4], stripes in zebrafish [5], and dorso-ventral patterning in *Xenopus* larvae [6]. At a much broader scale, single-cell transcriptomics and lineage tracing techniques have made it possible to map the diversity of forms of extant multicellular organisms [7]. Here, we ask about the *limits of diversity* that development can generate. And reciprocally, we ask what is common among all organisms that undergo development.

Biological development is modular [8], and its outcome rests on gene regulation that is switch-like, rather than continuous [9, 10]. Keeping this in mind, we constructed a generative model of development with three basic ingredients: asymmetric cell division, signaling, and gene regulation [11]. Although much is known about the detailed molecular machinery of development [12], naturally, these details come from studies on a few model organisms. We choose to not include all these important particular features in our model for the sake of efficiently and systematically sampling a broad space of developmental schemes. Nonetheless, our model is capable of expressing specific examples of known developmental pathways, which we demonstrate using the *Drosophila* segment polarity network analyzed in [9].

We encode organisms in our model as lineage graphs, which show differentiation trajectories of the various cell types in the organism. Traditionally, mathematical models in the literature elucidate developmental mechanisms responsible for known differentiation trajectories [13]. Here, we take the *inverse* approach, and at a much broader scale, we sample across millions of biologically plausible developmental rules and map out the lineage graphs they produce. We purposely do not include selection in the model since it is almost impossible to conjecture and quantify all potential selection factors, such as efficiency, robustness, evolvability, and their intertwined fitness effects on the developmental program. Instead, we sample developmental rules *uniformly* to provide an extensive chart of all possible programs without weighing their relative advantage. Our approach allows us to identify emergent properties that arise from combinations of the ingredients of biological development. We anticipate that such properties are likely to be universal, regardless of the selective pressures faced by these organisms.

By tuning just three biologically meaningful parameters—which control signaling, cellular connectivity, and cell division asymmetry—our model produces a rich collection of organisms with diverse cell type lineage graphs, ranging from those with a single cell type to organisms with close to a hundred cell types. Given the coarse-grained nature of the model, we do not expect model-generated organisms to resemble real organisms in all aspects. Instead, we examine and find hallmarks of multicellular organisms which originate from the fundamental features of development included in the model.

Notably, tree-like lineage graphs are rare in our model. This could indicate that, contrary to popular belief, lineage graphs of real organisms are not tree-like; they are more likely to be directed acyclic graphs (DAGs). Additionally, an unanticipated outcome of our model is that most organisms we generate are capable of whole-body regeneration. Our result supports the hypothesis that regeneration is an epiphenomenon of development, rather than a function that evolved separately [14]. The model also produces concrete predictions, and we discuss how these predictions can be experimentally tested on animals like *Planaria* and *Ascidia*, which are well-known models of animal regeneration [15, 16].

## Generative model of development

Organisms in the model contain genomes with *N* distinct genes. By “genes,” we refer not to single genes, but to gene regulatory modules that control cellular differentiation [17]. These genes encode for cell fate *determinants*. In different cell types of an organism, different sets of determinants can be present (1) or absent (0). We represent a cell state as a *N*-length binary string. For example, for *N*=3, a cell in state *C*=[101] contains determinants 1 and 3 but not the determinant 2. (In Additional file 1: Section 1.1, we demonstrate how we can also use “determinants” to encode spatial information using the well-known *Drosophila* segment polarity network as an example.)

**Fig. 1.**
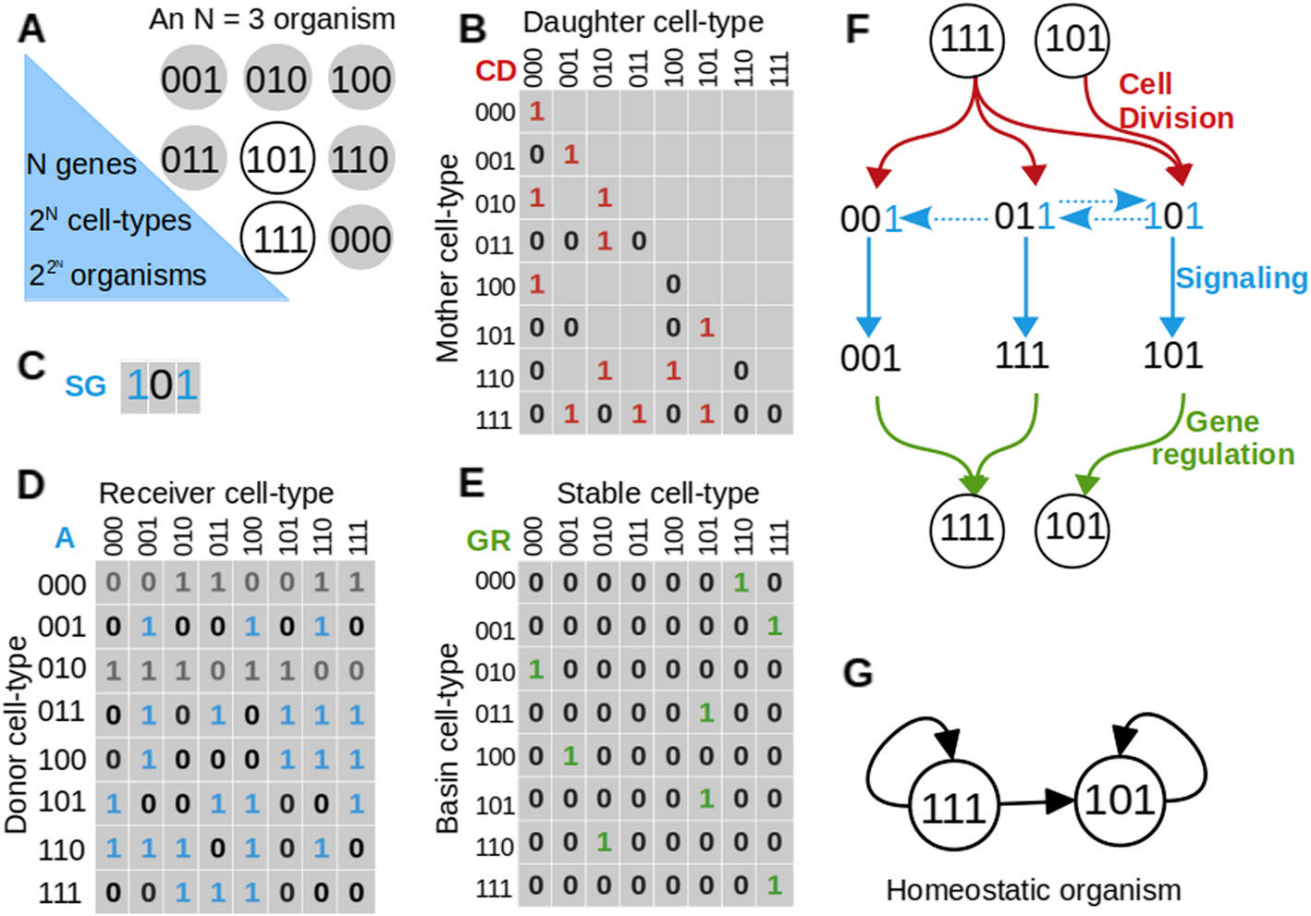
Generative model. **a** An organism with *N* = 3 genes and two cell types. Circles represent all possible cell types. The organism is composed of cell types represented by white circles and does not contain the gray cell types. Binary strings written inside the circles represent the presence (1) or absence (0) of determinants in those cell types. **b**–**e** The rules for the development of the organism in **a**. **b** Cell division matrix CD. In the model, daughter cell types cannot contain determinants not present in the mother cell type; therefore, in the figure, such positions in CD are represented by empty boxes. For all *j* such that CD(*i*,*j*)=1, cell type *i* produces cell type *j* upon cell division. **c** Signaling matrix SG. Determinants 1 and 3, which are labeled in blue, act as signaling molecules. **d** Signaling adjacency matrix *A*. *A*(*i*,*j*)=1 implies that cell type *j* receives all signals produced by cell type *i*. The rows for cell types [000] and [010] are grayed out, since these cells produce no signaling molecules. **e** Gene regulation matrix GR. GR(*i*,*j*)=1 implies that cell type *j* is a stable cell type, and cell type *i* maps to cell type *j*. **f** Schematic of “organismal development” in the model. All cell types synchronously undergo cell division according to CD; the daughter cells exchange signals according to SG and *A*, and cells respond to signals through gene regulation according to GR. The process repeats until it reaches a steady state. Here, we show how the homeostatic organism in **a** is obtained using the developmental rules matrices in **b**–**e**. **g** Lineage graph of the homeostatic organism in **a**

Cell types are ordered according to standard binary ordering, i.e., the cell [101] can equivalently be written as *C*_5_. We only look at whether a given cell type is present or absent in organisms, rather than the number of cells of any given cell type. Therefore, since each of the *N* determinants can be either 1 or 0, there are at most 2^*N*^ distinct cell types in an organism and $2^{2^{N}}$ cell type compositions for organisms (Fig. 1a). Note that the number of distinct organisms is larger than $2^{2^{N}}$, since different organisms may have the same set of cell types but distinct lineage graphs (Fig. 1g).)

We represent development as a repeated sequence of cell division, intercellular signaling, and gene regulation.

### Cell division

Cells in the model undergo asymmetric cell division, where daughter cells inherit determinants from the mother cell in an asymmetric manner. That is, a determinant that is present in the mother cell may not be inherited by all its daughter cells due to unequal or insufficient partitioning during division [18]. In the model, asymmetry of cell division is controlled by the parameter *P*_asym_∈[0,1], which is the probability that a daughter cell does not inherit a given determinant from the mother cell; *P*_asym_=0 implies symmetric division, where all daughter cells inherit all determinants from the mother cell; and at *P*_asym_=1, no daughter cell inherits any determinants from the mother cell.

Although in real multicellular organisms, a single cell only divides into two daughter cells; a single cell type may represent a population of cells, which need not all behave in the same way [19, 20]. We capture this heterogeneity by allowing cells in our model to divide into more than two types of daughter cells. For any given organism in the model, we predetermine the sets of daughter cells produced by any cell type randomly according to *P*_asym_ and encode this in a binary matrix CD (Fig. 1b).

In real organisms, asymmetrically segregating determinants actively influence functionality of cells. Some determinants modulate the response of cells to signals, most famously, the protein Numb, which is an inactivator of Notch signaling, is asymmetrically segregated during the division of neural, muscle, and hematopoeitic stem cells [18]. Other asymmetrically segregating proteins act as signaling molecules themselves, for example, the protein Dll1 segregates asymmetrically during neural stem cell division and is sent as a signal to neighboring quiescent neural stem cells [21].

### Signaling

The number of distinct signaling molecules in an organism is controlled by the parameter *P*_sig_∈[0,1], which is the probability that any determinant is a signaling molecule. Parameter *P*_adj_∈[0,1] controls signal reception; a cell type *C*_*i*_ can receive signals from a cell type *C*_*j*_ with probability *P*_adj_. Additionally, during simulations, cells can only receive signals from other cell types present in the same time step, and recipient cells receive all the signals produced by donor cells. As in the case of cell division, for each organism, the set of signaling molecules, and the pairs of cells that are allowed to exchange signals are predetermined and stored in a binary vector SG and a binary matrix *A*, respectively (Fig. 1c, d).

### Gene regulation

The combination of determinants inherited by a daughter cell during cell division and those received as signals from other cells present in the same time step together regulate gene expression in this cell. We model gene regulation as random Boolean networks (RBNs) [22]; here, the transcriptional states of genes depend on each other through arbitrarily complex Boolean rules. Updates in gene states lead to updates in the states of determinants, which in turn result in updates in cell types. In this scheme, some cell states update to themselves (stable cell type), and other cell states ultimately update to one of the stable cell states; that is, they lie in the basin of a stable cell type. Note that a cell type in the model need not be a fixed point (single cell state) of the gene regulatory network, it can also be an oscillation (multiple cell states) [23]. In the latter case, the cell type is represented by all cell states that are part of the oscillation. Here, we are only concerned with the set of cell states in the stable state, and not with the sequence of cell states in oscillations.

In the model, instead of encoding RBNs explicitly, we describe gene regulation directly as the set of stable cell types and their basins. Stable cell types and cell states in their basins are both chosen uniform randomly, as described in detail in the “Methods” section. Generally, gene regulatory networks with higher *N* have more stable cell types, but basin sizes, on average, remain small (1–2 cell states) across all *N* (Additional file 1: Figure S3). For each organism, we predetermine its gene regulation and encode it in a binary matrix GR (Fig. 1e). The matrices CD,SG, *A*, and GR should be viewed as summaries of all processes that determine cell fate during the lifetime of an organism, and a simulation of the model represents a recapitulation of all these events. Our model is deterministic; for a given organism, the matrices CD,SG, *A*, and GR are independently generated, and they remain fixed for the rest of the simulation. The model is also synchronous; all cell types in the organism divide simultaneously, after which the developing organism is composed only of all daughter cells produced in this step. These daughter cells simultaneously exchange signals, in response to which the states of all the determinants, in each daughter cell are updated simultaneously according to GR (Fig. 1f). A time step in the model represents a single repeat of cell division, signaling, and gene regulation.

The process of development ends when the set of cell types in a developing organism repeats itself. We call this set of cell types the steady state of the organism, and the number of time steps between two repeats the period of the steady state. Since this is a finite and deterministic system, starting from any initial condition, such a steady state can always be reached. We call period 1 steady states *homeostatic organisms* (Fig. 1f). Although organisms with complex, period >1 life cycles, such as land plants with alternation of generation [24] exist in nature, in this study, we focus on homeostatic organisms. We represent homeostatic organisms as their *cell type lineage graphs* (Fig. 1g). The nodes of this graph represent cell types in the homeostatic organism, and directed edges represent lineage relationships between these cell types. Let some cell types *A* and *B* in a homeostatic organism be represented by nodes *V*_*a*_ and *V*_*b*_, respectively, in its lineage graph. Then, there is an edge from *V*_*a*_ to *V*_*b*_ if one of the daughter cells of *A* gives rise to *B* after one round of cell signaling and gene regulation.

In the model, all the cell state transitions that lead to the homeostatic organism starting from an initial cell type represent the process of development, while the homeostatic organism itself represents the product of development. In this work, we study the properties of these homeostatic organisms and their lineage graphs.

## Results

### Homeostatic graphs span a large range of sizes

**Fig. 2.**
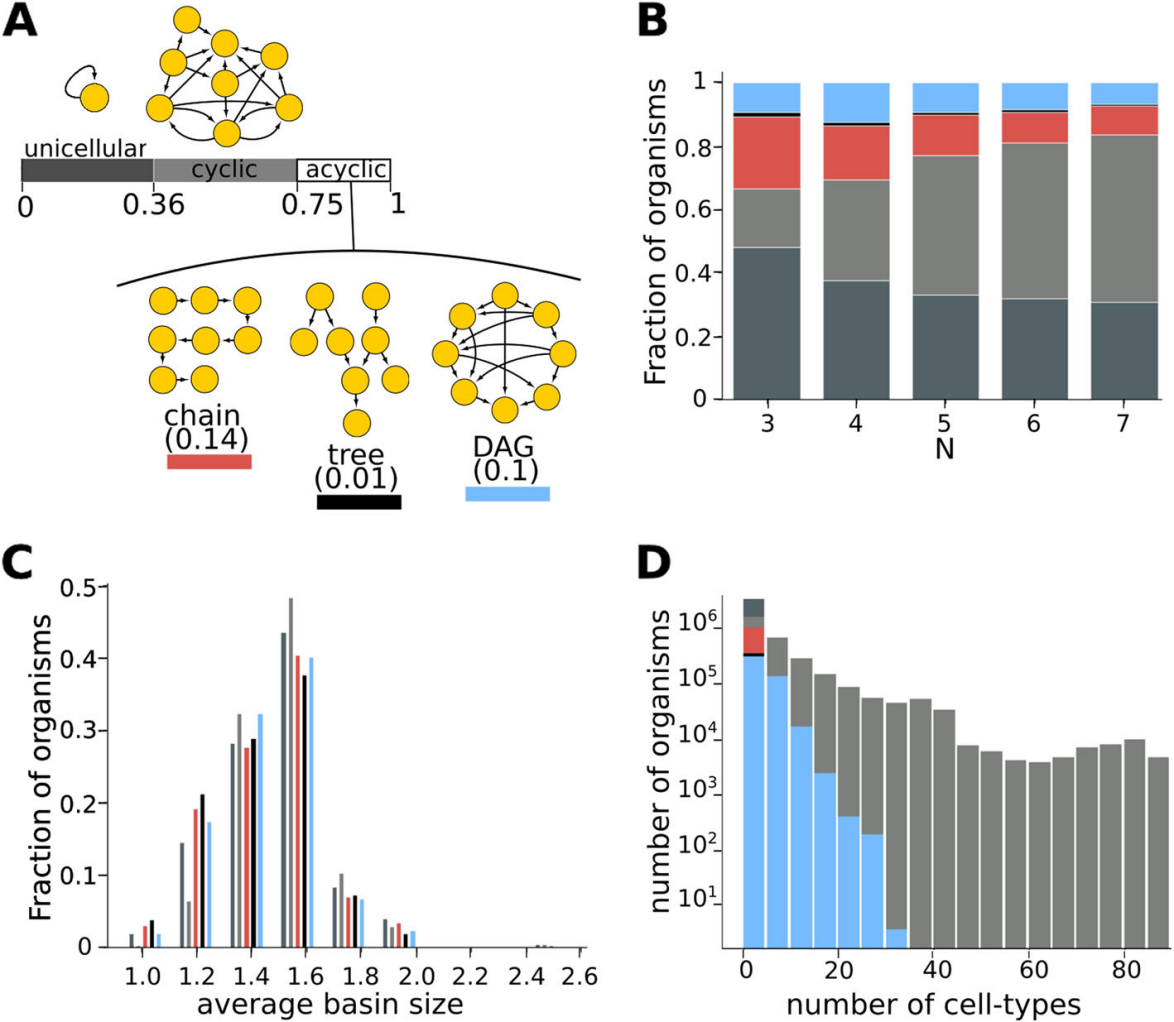
Diversity of lineage graphs. A total of 4,852,994 graphs were used to generate the following figures. **a** Abundance of lineage graph topologies. The bar represents the fraction of graphs that have a given topology. Graphs given along the bar are examples of model-generated lineage graphs. Parameters (*N*,*P*_asym_,*P*_sig_,*P*_adj_) used to generate these graphs: unicellular (3, 0, 0, 0), cyclic (4, 0.2, 0, 0.5), chain (5, 0, 0.4, 0.9), tree (6, 0, 0.4, 1), and DAG (4, 0.1, 0, 0.4). Colors indicate lineage graph topology: unicellular: dark gray; cyclic: light gray; chain: red; tree: black; and DAG: blue. **b** Stacked histogram for topologies of lineage graphs obtained with different *N*. Heights of colored blocks represent the proportions of corresponding topologies. **c** Distribution of basin sizes in the gene regulatory networks across different lineage graph topologies. For any given topology, the height of bars indicates the fraction of lineage graphs of that topology that were obtained using a gene regulatory network whose average basin size is given along the horizontal axis. **d** Stacked histogram showing distribution of number of cell types in homeostatic organisms with lineage graphs of various topologies. See also Additional file 1: Figure S9

We looked at millions of homeostatic organisms, spanning systems with *N*=[3,4,5,6,7] number of genes (Additional file 1: Figure S3a). 99.88% of these homeostatic organisms had lineage graphs with a single connected component. In the following, we describe the lineage graphs of these single-component homeostatic organisms. While a majority of graphs in our data are small (1–5 cell types), the largest graphs have 89 cell types (Fig. 2d, Additional file 1: Figure S4a). Across different organisms, the number of cell types in lineage graphs increases with the diversity of daughter cell types produced (Additional file 1: Figure S4b,c) and decreases with the level of signaling (Additional file 1: Figure S4d,e,f). Naturally, the number of edges in lineage graphs increases with the number of cell types, but this increase is slower than that expected for simple random graphs (Additional file 1: Figure S5).

### Diversity of lineage graph topologies and the dearth of tree-like lineage graphs

Paths in a lineage graph represent differentiation trajectories of the organism’s cell types. Here, we classify lineage graphs into five topologies, each of which contain qualitatively different paths: (i) unicellular graphs, (ii) cyclic: multicellular graphs that contain some cyclic paths, (iii) chains: acyclic graphs with no branches, (iv) trees: acyclic graphs with branches, and (v) other directed acyclic graphs (referred to here simply as DAGs): acyclic graphs with branches and *links*, which are edges that connect different branches. Links represent the convergence of multiple cell lineages to the same terminal cell type. We ignore self-edges during lineage graph classification.

In our data, unicellular graphs are the most abundant (36%). Acyclic graphs (chains, trees and DAGs) comprise about 25% of our graphs. Of these, trees are the rarest (1% across all graphs), and chains are the most abundant (14.3% across all graphs) (Fig. 2a,b). We find that even after including “acyclized” versions of cyclic graphs in our analyses—i.e., where we merge all nodes belonging to each strongly connected component into single nodes—trees are still the rarest graphs (Additional file 1: Figure S18). Although all topologies are spread widely across parameter space, different topologies are enriched in different regions of parameter space (Additional file 1: Figure S6) and differ in their graph size distributions (Fig. 2d, Additional file 1: Figure S9). Particularly, acyclic graphs are more likely than cyclic graphs to be generated using gene regulatory rules where stable cell types have smaller basins (Fig. 2c). While the lineage graph topologies obtained at any parameter value do vary depending on *GR*, at the sample sizes used in this study, details of *GR* do not influence the distribution of lineage graph topologies (Additional file 1: Figure S7)). To a large extent, these topologies can be characterized by their in-degree and out-degree distributions (Additional file 1: Figure S8a,b). However, we find that acyclic graphs are slightly more enriched in our data than in randomized graphs with the same in-degree and out-degree distributions (Additional file 1: Figure S8c,d).

### Real homeostatic lineage graphs

**Fig. 3.**
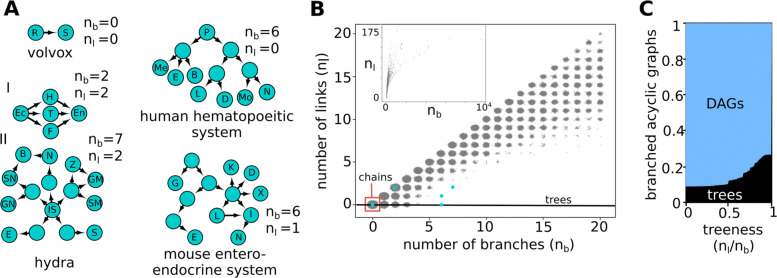
Lineage graphs of real organisms. **a** Lineage graphs from real organisms: Volvox: R = reproductive cell, S = somatic cell [25]; human hematopoietic system: P = progenitor cells, Me = megakaryocytes, E = erythrocytes, B = basophils, L = lymphocytes, D = dendritic cells, Mo = monocytes, N = neutrophils [26]; hydra: Ec = ectoderm, En = endoderm, IS = interstitial cell, H = hypostome, T = tentacle, F = foot, E = egg, S = sperm, GN = ganglion neuron, SN = sensory neuron, B = battery cell, N = nematocyst, Z = zymogen granule cell, GM = granular mucous granule cell, SM = spumous mucous granule cell [27]; and the mouse entero-endocrine system: G = goblet cell, E = EC cell, K = K cell, D = *δ* cell, X = X cell, L = L cell, I = I cell, N = N cell [28]. **b** Scatter plot of the number of branches versus the number of links for acyclic lineage graphs with *n*_*l*_ and *n*_*b*_<= 20. The inset shows the *n*_*l*_ versus *n*_*b*_ scatter plot for all acyclic graphs. Noise has been added to points to make density of points more apparent. 1,217,108 graphs were used to generate this plot. **c** Relaxed definition of treeness: the *x*-axis represents *n*_*l*_/*n*_*b*_, our measure of a threshold for “treeness.” *n*_*l*_/*n*_*b*_=0, represents the traditional, strict definition of trees, whereas at *n*_*l*_/*n*_*b*_=1, all branched acyclic graphs are considered trees. At intermediate values, the fraction of graphs labeled as trees increases slowly. A total of 521,136 graphs were used to generate this plot

Under normal circumstances, cellular differentiation is expected to be irreversible; therefore, we analyzed the acyclic graphs of our model in more detail. We characterize acyclic graphs on the basis of two features: number of *branches* (*n*_*b*_ = the total number of paths from root nodes to leaf nodes in the graph – 1) and number of *links* (*n*_*l*_ = number of edges in the graph – number of edges in the maximal spanning tree of the graph) (Fig. 3b, see also Additional file 1: Figure S11). While for all chain type graphs, both *n*_*l*_ and *n*_*b*_ equal 0, for all tree type graphs, *n*_*l*_=0 and *n*_*b*_>0. In Fig. 3c, we see that the fraction of trees in our data remains low even if we relax the above definition of trees and allow *n*_*l*_ to be non-zero. Even considering a threshold “treeness” as high as *n*_*l*_/*n*_*b*_=0.5, we find that the fraction of tree-type graphs in our data only increases from 0.01 to 0.013% (see also Additional file 1: Figure S18). Therefore, in the following, we use only the strict definition of trees, *n*_*l*_/*n*_*b*_=0, as it does not affect our main conclusions.

We compare the model-generated lineage graphs with examples of real lineage graphs collected from the literature (Fig. 3a). While many reports of lineage graphs exist in the literature, especially owing to recent single cell transcriptomics studies, most graph reconstruction algorithms are biased to report trees [29, 30], and hence are not used in this study. We include only lineage graphs constructed in an unbiased way in this comparison.

The currently available real lineage graphs are still too few to statistically infer their features. Nevertheless, from this small sample, it appears that real lineage graphs, especially mammalian ones, contain more branches and fewer links than model-generated graphs. This could indicate that the model is not sufficient to fully capture lineage graph topologies, or alternatively, that additional, unbiased real lineage graph reconstructions are required for rigorous analysis.

### Homeostatic organisms contain pluripotent cells

We next looked at functional properties of model-generated homeostatic organisms; in particular, we tested whether these organisms can regenerate using pluripotent cells. We define a pluripotent cell as any single cell type which develops into a homeostatic organism using the same rules (GR,CD, *A*, and SG) used to generate this organism in our data. We find that in 92.6% of organisms with acyclic lineage graphs (and 97% of all organisms), there is at least one pluripotent cell type which is a part of the homeostatic organism. Since homeostatic organisms are stable products of the process of development, we consider them to be *adult organisms*. And we call pluripotent cells which are part of homeostatic organisms *adult pluripotent cells*.

**Fig. 4.**
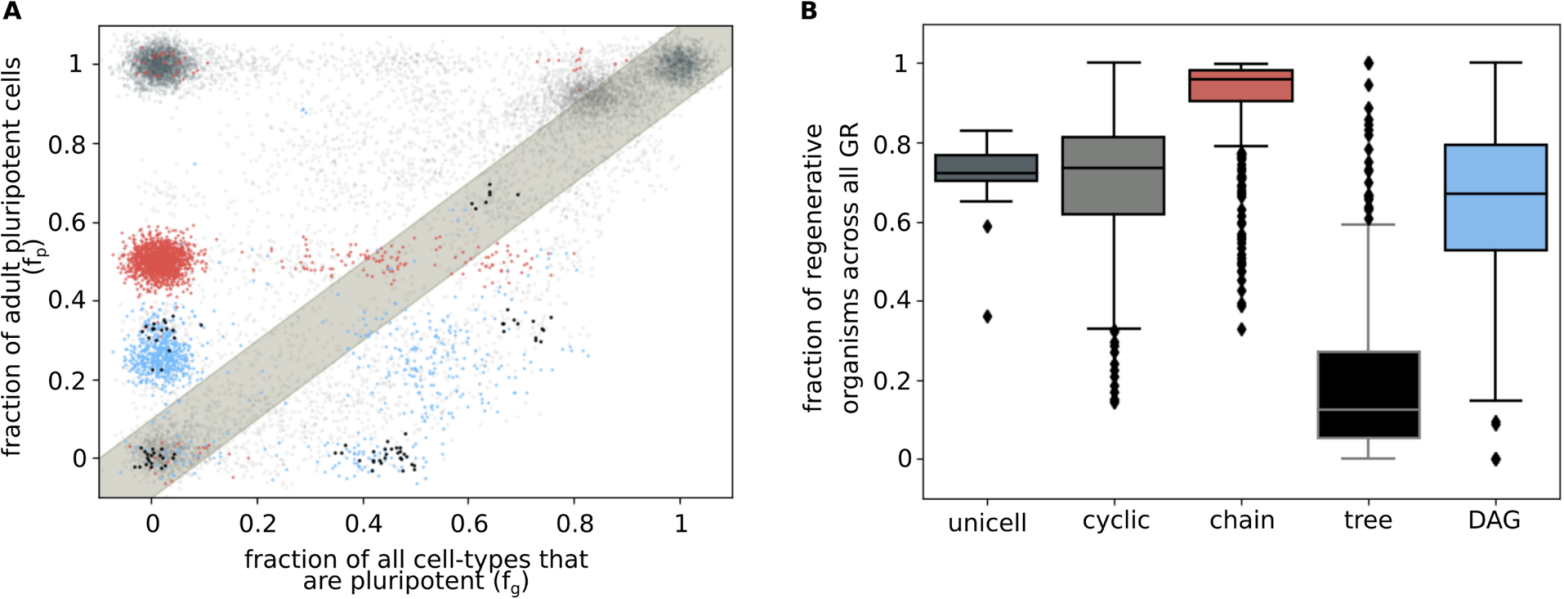
Regenerative capacity. **a** Scatter plot showing the regenerative capacity for all *N*=7 organisms generated with a fixed gene regulation matrix GR using different matrices CD, *A*, and SG (for cell division, cellular adjacency, and signal transduction, respectively). Each point represents an organism. The *x*-axis is the fraction of all cell types that are pluripotent (*f*_*g*_). The *y*-axis is the fraction of cell types in the organism that are *adult pluripotent* cells (*f*_*p*_). Noise has been added to the position of points to make their density more apparent. Colors of points indicate the topology of their lineage graphs (as in Fig. 2): unicellular: dark gray; cyclic: light gray; chain: red; tree: black; DAG: blue. Points above the gray band are regenerative organisms (with *f*_*p*_/*f*_*g*_>1). A total of 13,177 graphs were used to generate this plot (see also Additional file 1: Figure S12). **b** Box plot of proportion of regenerative graphs of different topologies across all organisms in the data (see also Additional file 1: Figure S13a). For each GR used in our data, for a given graph topology, we looked at the fraction of graphs with regenerative capacity >1 (equivalent to the fraction of points of a certain color that occur above the gray band in **a**). Boxes represent quartiles of the data set. Lines inside the box show the median, while whiskers show the rest of the distribution. Outliers are shown as diamonds. A total of 4,852,994 graphs were used to generate this plot

Among real organisms, there exist both organisms which contain adult pluripotent cells (e.g., planaria) and those that do not (e.g., humans). In our data, in 82.9% of acyclic lineage graphs (73.3% of all graphs), pluripotent cells are more likely to be part the adult organism than not (Fig. 4a).

Evidently, organisms that contain adult pluripotent cells are more disposed to be regenerative than those that do not. We therefore measure *regenerative capacity* of an organism as the fraction of cell types in the organism that are adult pluripotent cells (*f*_*p*_) divided by the fraction of all cell types (irrespective of whether it is a part of the adult organism or not) that are pluripotent (*f*_*g*_). We call an organism *regenerative* if its regenerative capacity is greater than 1. We find that regenerative capacity differs among different topologies (Fig. 4b, Additional file 1: Figure S13a). Notably, while most chains are regenerative, most tree-type graphs have very low regenerative capacity (see Additional file 1: Figure S19 for regenerative capacities using relaxed definition of trees). More generally, this distribution of regenerative capacities serves as an example of correlations between high-level functions of organisms with their lineage graph topologies. Such associations could form the basis for natural selection favoring certain topologies over others in real multicellular organisms.

### Signal transduction influences regeneration trajectories

The high level of adult pluripotent cells in homeostatic organisms is surprising, since cell fates within the organism are constrained due to signaling, and cells taken out of the context of signaling from other cell types in the organism are not expected to regenerate the other cell types.

**Fig. 5.**
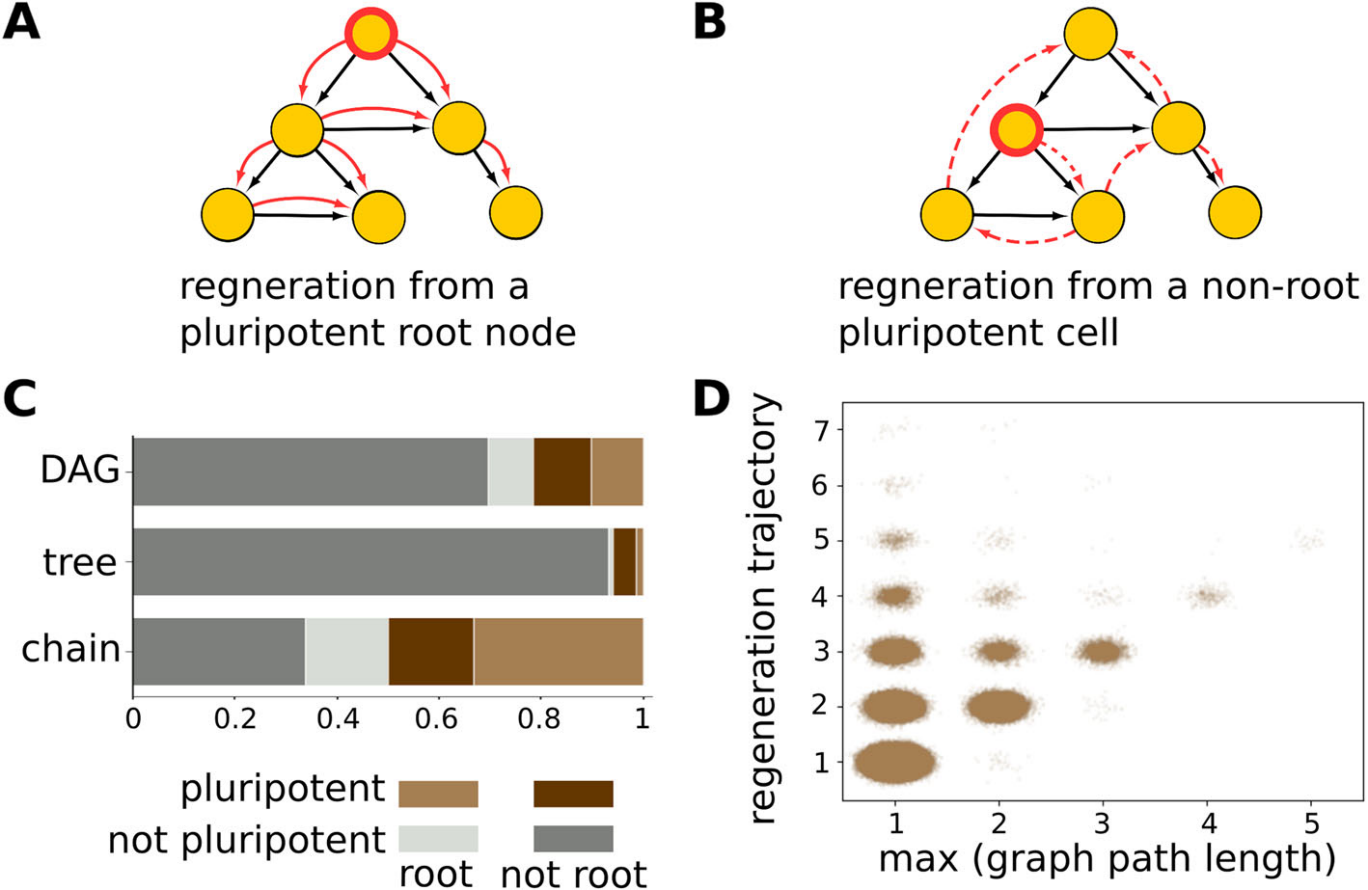
Regeneration trajectories. **a**, **b** Schematics of regeneration trajectories.Yellow circles represent cell types in a homeostatic organism, and black edges represent lineage relationships. Adult pluripotent cells are outlined in red. Red edges represent lineage relationships between the organism’s cell types during regeneration. **a** Here, the root node is the only adult pluripotent cell and regeneration trajectories exactly match the paths in the lineage graph. In cases where signaling does not play a role in determining cell fate, all regeneration trajectories are of this kind. **b** Here, a non-root node is a pluripotent cell, and therefore, necessarily, regeneration trajectories cannot be perfectly aligned with paths in the lineage graph. In these cases, signaling is definitely involved in governing cell fates. Dashed red edges imply that cell-types other than those present in the homeostatic organism may be produced during regeneration. **c** Stacked histograms for cell types of different categories pooled from organisms with different lineage graph topologies. Different cell type categories are represented with different colors. Non-pluripotent cells are represented in grays; root nodes: light gray; not non-root nodes: dark gray. Adult pluripotent cells are represented in colors: root nodes: light brown, non-root nodes: dark brown. Heights of colored blocks represent the proportions of corresponding cell types. A total of 1,217,108 graphs were used to generate this plot. **d** Scatter plot showing the maximum path lengths across lineage graphs vs the lengths of regeneration trajectories from pluripotent root nodes for acyclic lineage graphs that contain them. Each point represents an organism. Noise and transparency has been added to the position of points to make their density more apparent. A total of 699,986 graphs were used to generate this plot

In order to test the mechanism of regeneration in the model, we asked two questions: (1) how much does cell fate in the organisms depend on signaling? and (2) what do regeneration trajectories look like? If regeneration does not depend on signaling, cell types should exactly retrace their paths in the homeostatic lineage graph, irrespective of the presence of other cell types (Fig. 5a).

To answer the first question, we define the fate of a cell type *C* in a homeostatic organism as the set of all cell types that receive an edge from cell type *C* in the lineage graph of the organism. Note that the above definition refers to a *proximal* cell fate, where only the immediate descendants of a cell type are considered. We call a cell type *independent* if it has the same cell fate when taken out of the homeostatic organism, as it does within the organism. We find that across all parameter regions, homeostatic organisms are enriched in independent cell types (Additional file 1: Figure S14). While about 32% of all independent cells, pooled from all acyclic graphs, are adult pluripotent cells, 90% of all adult pluripotent cells are independent (Additional file 1: Figure S15). That is, in the model, proximal cell fates, especially those of adult pluripotent cells, are likely to be independent of signaling.

To resolve the second question, note that if regeneration trajectories recapitulate the homeostatic lineage graph, (a) only *root nodes*, which are nodes from which all other nodes of the lineage graph are reachable, can be adult pluripotent cells, and (b) regeneration trajectory lengths must match the longest of the minimum path lengths from the root node to any of the leaf nodes in the lineage graph.

Now, necessarily, there can be no more than a single root node in an acyclic graph. Some acyclic graphs, for example, convergent trees (Additional file 1: Figure S10), even lack root nodes. In our data, only 7.4% acyclic graphs lack a root node, and of these, 85.4% indeed lack adult pluripotent cells. And 90.4% of the time, in rooted acyclic graphs, the lengths of regeneration trajectories starting from pluripotent root nodes do match the maximum path length in the respective lineage graphs (Fig. 5d). But, among these rooted, regenerative acyclic graphs, only 62.9% have root nodes that are pluripotent. Overall, 41.6% of adult pluripotent cells are *not* root nodes (Fig. 5c). Regeneration trajectories starting from such non-root adult pluripotent cells are bound to be different from paths in the homeostatic lineage graph (Fig. 5b), and therefore must involve signaling. Consistently, lineage graphs with non-root adult pluripotent cells are more likely to be generated at higher values of *P*_sig_ and *P*_adj_ than lineage graphs with only root pluripotent cells (Additional file 1: Figure S16).

To summarize, although cell fates for most cell types in organisms are independent of signaling, almost half the time, signal exchange definitely plays a role in regeneration, and regeneration trajectories of organisms are different from paths in their homeostatic lineage graphs. Parallels to these results can be seen in the development and regeneration of ascidians, which undergo “mosaic development,” where cell fates are independent of cellular context and are determined by *autonomous specification* [31]. But the level of plasticity during asexual budding (which can be seen as a form of programmed whole body regeneration) in colonial ascidians, which are model organisms both for mosaic development and regeneration, points to a “non-mosaic” mode of regeneration [32].

## Discussion

The process of development and its molecular mechanism are inherent in all *metazoans* and in all plants [3]. It is therefore difficult to design experiments that could distinguish between emergent traits associated with development and traits that have evolved on top of it. Here, we have developed a minimal model where we can look at development in the absence of complications due to cross-talk with other biological processes. In our model, we include only those ingredients of development that are shared across all multicellular organisms, while not ascribing any particular form or mechanism to these processes. This allows us to identify universal traits that are inherent to development, regardless of the details of the process, or the distinct selective pressures different organisms may be subject to. We see such a prominent emergent trait in our model: ability of *whole body regeneration* (WBR) through pluripotency. Concurrently, WBR, although absent in many animals, such as mammals, is widely spread across basal metazoan phyla.

Note that such basic traits can still be subject to selection through regulatory processes on top of the key ingredients of development. Below, we discuss major assumptions and limitations of our model and contrast these with mechanisms that occur in biological organisms.

- *Considerations of space and time:* Spatial arrangement of cells and cell movements are important and highly regulated aspects of development. Two cells having the same cell type but occupying different niches are likely to receive different sets of signals and therefore are expected to behave differently [12]. Cells are also conjectured to lose potency as development progresses, such that cell types present in later stages of development are likely to give rise to fewer cell types than those present in the earlier stages [33]. Such a process can be captured in the model by using developmental rules endowed with a *flow* such that cell types appearing later in development have lower potency. The final outcome of development is highly likely to be affected in those organisms where such a mechanism operates.
  Although in the current study, due to our focus on widely exploring asymmetric cell division, signaling, and gene regulation, we do not explore the important aspects of space and time; we provide a recipe for how they can be included in future studies: in Additional file 1: Section 1.1, using the example of the *Drosophila* segment polarity network; and we show how space can be encoded within the framework of the model. Developmental time can be included using a similar approach.
- *Independent processes:* Cell division, signaling, and gene regulation are treated as independent processes in the model. This is likely to be false in real animals. Primarily, this implies that not all regions of parameter space explored in this work are biologically feasible. In particular, cells in the model follow a simple program for asymmetric cell division that is intrinsic to cell types. But extrinsic control of asymmetric cell division, involving cues from surrounding cells, does occur in animals [18]. Extrinsic control of asymmetric cell division could lead to a decrease in the independence of cell fates on cellular context which we see in the model, which could affect regenerative capacity.
- *Chemical signaling:* In our model, signal recognition is based on identities of the donor and the recipient cells. In contrast, in real organisms, cells contain receptors that recognize signal molecules, rather than recognizing the donor cells that produce those signals. Firstly, since there are fewer kinds of signal molecules than there are cell types, it is likely in this chemical recognition scheme that a cell type will receive the same set of signals even if some other cell types in the organism are changed. That is, cell fate is likely to be even more robust to changes in cellular context than the present model. In our model, cell fates of most pluripotent cells are independent of cellular context. Therefore, a version of the model with chemical recognition is also likely to yield regenerative organisms.
- *Other schemes:* In the current model, we use the following scheme of development: cell division, followed by signaling among daughter cells and gene regulation in response to signals exchanged. But there are other reasonable schemes which can also be considered. For example, a scheme where cell division is followed by an additional step of gene regulation before signal exchange is also plausible. In the current model, daughter cells contain subsets of the contents of the mother cell, and in this sense are more similar to each other than to daughters of other mother cells. Therefore, in the current scheme, signals received from a sister cell are likely to be less *effective* in changing cell state than are signals received from other daughter cells. Gene regulation right after cell division would lead to a diversification of daughter cells, which is therefore likely to increase the level of effective signaling among daughter cells. In our model, level of signal exchange is controlled by *P*_adj_, and we find that pluripotency increases, albeit modestly, with *P*_adj_ (Additional file 1: Figure S13e). Therefore, we expect that switching to this other, more elaborate scheme of development would still lead to high regenerative capacity.
- *Additional parameters:* The effect of processes such as asynchronous gene state updates [34] and time delays involved in transfer of information about gene state updates [35] has been tested on the *Drosophila* segment polarity network and found to have interesting effects on the robustness of phenotypes. Such processes could add to the richness of lineage graphs we obtain from our model, but come with the cost of additional parameters, which would limit the breadth of the sampling.
- *Cell death:* Cells in the model do not die. Not including cell death in the model results in lineage graphs where each node has at least one out-edge. We anticipate that including cell death would reduce the number of cycles in lineage graphs, leading to an increase in the proportion of acyclic graphs (Additional file 1: Figure S17). Since regenerative capacity is linked to lineage graph topology, cell death could be an important factor in determining regenerative capacity. We also anticipate that including cell death could add a sense of developmental time to the model.

Although here we only provide intuitive arguments for what alternate versions of the model might yield, the framework of the model is easily amenable to manipulations, and differently constructed versions can be tested in the future.

The present model makes several predictions regarding general features of development and multicellular organisms. It suggests that the presence of *adult pluripotent cells* should be a widespread trait in multicellular life forms. In plants, we are already aware of pluripotent cells in the root and shoot meristems. But among animals, a wider investigation of regeneration and its mechanisms will be required to test this idea. A recent example of such a study is [36], where the authors test the ancestral nature of regeneration in *Nemertaean* worms, which are not classical model organisms.

The distribution of acyclic lineage graph topologies in our data reflect the complexity and diversity of forms of multicellular animals that biological development is expected to produce. Small (2-node) *chains* are the most abundant acyclic lineage graphs in our data (Fig. 2d, Additional file 1: Figure S9c). In line with this, the simplest multicellular organisms, such as *Volvox carteri* [25], an alga which evolved multicellularity only recently, has a chain-like lineage graph. Interestingly, some cyanobacteria, such as *Anabaena spaerica* [37], which display multicellularity during nitrogen starvation, also have chain-like lineage graphs.

*Tree-type lineage graphs are rare* in our data (Fig. 2a, b), tend to be small, and convergent rather than divergent (Additional file 1: Figures S9e, S10). This could indicate one of two things: This could imply that lineage graphs of complex organisms are unlikely to be tree-like. Our data suggests that they are more likely to be directed acyclic graphs (DAGs), i.e., organisms have higher levels of trans-differentiation than expected (Fig. 3b, Additional file 1: Figure S11). Or, it could mean that more complex regulation, on top of the ingredients of this model, is at play in real organisms which lead to complex tree-like lineage graphs. A perhaps presumptuous, but interesting possibility is that tree-like lineage graphs were selected for because of their low regenerative capacity. There exist arguments and speculation over whether mammals, among other animals, selectively lost the ability to regenerate, and why [38].

These questions surrounding the topologies of lineage graphs are likely to be resolved very soon in the future, given the rapid developments in single-cell transcriptomics technology. A notable recent study is that of Plass et al. [39], where they assemble the whole organism lineage graph for *Planaria*. A possible hurdle comes from the fact that in such studies, lineage relationships cannot be directly accessed and are instead inferred using distances between cellular transcriptomes obtained at different times. Moreover, current methods for lineage reconstruction using single-cell transcriptomics data are not unbiased; in [39], although lineage reconstruction yielded a complex graph, the authors highlight the best supported spanning tree of this graph. Current lineage reconstruction methods work best if a particular topology for lineage graphs is already anticipated, and most methods are designed to only find chains and trees [29, 30]. In contrast, a study by Wagner et al. [40], where single-cell transcriptomics is used in conjunction with cellular barcoding, provides an example of a lineage reconstruction method which is unbiased towards particular topologies. In agreement with our result, the authors of this study found that zebrafish development is best represented by a DAG.

Lastly, we discuss how certain predictions of our work can be experimentally tested. Our work suggests that in colonial ascidians, which reproduce asexually using a variety of budding structures, the pattern of regeneration should be “non-mosaic,” where regeneration trajectories do not recapitulate lineage trajectories in the homeostatic organisms. In contrast, our model suggests that in *Planaria*, where pluripotent c-Neoblasts appear to occupy the root node [39], regeneration trajectories are likely to reflect the homeostatic lineage graph. These predictions can be addressed by lineage reconstruction experiments that compare homeostatic lineage graphs with lineage graphs produced during regeneration of these organisms.

Our results also suggest that in organisms such as *Planaria* and perhaps colonial *Ascidia*, where regeneration is based on adult pluripotent cells [15, 16], these cells are likely to be independent of cellular context; that is, their proximal cell fates should not change when taken out of the body or transplanted to other cellular contexts. In *Planaria*, c-Neoblast independence could explain the coarse pattern of distribution of specialized neoblasts across the planarian body, and also why the distribution of specialized neoblasts produced does not depend on which organ is amputated [15]. Recent development of a method to culture neoblasts in the lab [41] make it possible to experimentally test neoblast independence.

## Conclusions

Development transforms single-celled zygotes into multicellular adults by combining three basic processes: asymmetric cell division, cell signaling, and gene regulation. Despite undergoing this common set of developmental processes, multicellular organisms display a huge variety of forms. In this work, we use a generative model of development to gauge the extent of possible diversity of multicellular forms. We explore the forms of “organisms” generated by our model in terms of their cell type lineage maps. Our data indicates that cell type lineage maps are unlikely to be tree-like, and instead that organisms are likely to undergo a much higher level of trans-differentiation than anticipated. Additionally, “organisms” generated in our model contain adult pluripotent cells and are thus likely to be capable of whole-body regeneration. This observation supports the view that whole body regeneration is an epiphenomenon of development. Regenerative capacity differs among organisms with different lineage graph topologies, tree-type lineage graphs having the lowest regenerative capacities. These differences could potentially serve as a basis for selection biased towards certain topologies. Our results also suggest that regeneration trajectories are likely to deviate from paths in the cell lineage graph and could involve cell types not present in the adult organism. To summarize, our work ties together the process of development and the phenomenon of regeneration and suggests many testable hypotheses, which can be addressed through experiments on well-established model organisms but, more importantly, through a wider sampling of cell differentiation trajectories across multicellular organisms.

## Methods

### Surveying the combinatorial space of developmental schemes

We considered organisms with *N*={3,4,5,6,7} genes. For each *N*, we have looked at {100,100,100,92,25} randomly generated gene regulation matrices (GR), respectively. For each GR, all values from $[0,0.1,0.2,\dots,1.0]$ were used for the parameters *P*_asym_,*P*_sig_, and *P*_adj_. A distinct set of developmental rules matrices (CD, *A*, and SG) was generated for each set of parameter values. And for each set of rules matrices, 10 randomly chosen cell types were used as initial conditions (in case of *N*=3, all 8 cell types were used). In all, we have looked at about ((100+100+100+92+25)×11^3^×10)≈5.5×10^6^ systems. A total of 4,858,643 of these converged within 1000 time steps into homeostatic organisms.

### Model details

All codes used to generate and analyze data are written in Python3.6 or Octave 5.2.0.

#### Asymmetric cell division

In our model, for any cell type *C*_*i*_, we generate different sets of daughter cell types *D*_*i*_ using the parameter *P*_asym_∈[0,1]; for any daughter cell type $D_{i_{i1}}\in D_{i},\forall k \leq N$,

$$\begin{array}{@{}rcl@{}} &&\text{if}\,\, \left(C_{i}(k) = 0\right)\,\, \text{then}\,\, \left(D_{i_{i1}}(k) = 0\right),\,\, \text{and}\\ &&\text{if}\,\, \left(C_{i}(k) = 1\right)\,\, \text{then}\,\, \left(D_{i_{i1}}(k) = {\text{Ber}}({P_{\text{asym}}})\right) \end{array} $$

We encode cell division in a binary matrix $\phantom {\dot {i}\!}{\text {CD}}_{2^{N} \times 2^{N}}$; CD(*i*,*j*)=1 if cell type *C*_*j*_∈*D*_*i*_, else CD(*i*,*j*)=0 (Fig. 1b).

#### Signaling

The probability that a gene in the model produces a signaling molecule is *P*_sig_∈[0,1]. Formally, let SG={0,1}^*N*^ be a binary vector. Then, gene *k* produces a signaling molecule if SG(*k*)=1, where SG(*k*)=Ber(*P*_sig_) (Fig. 1c). Let SG_*j*_={0,1}^*N*^ be the set of signals produced by cell type *C*_*j*_. For any gene *k*, SG_*j*_(*k*)=1 ⇔ (*C*_*j*_(*k*)=1)∧(SG(*k*)=1).

Parameter *P*_adj_∈[0,1] gives the probability of signal reception. We encode signal reception in a binary matrix $\phantom {\dot {i}\!}A_{2^{N}\times 2^{N}}$. Cell type *C*_*i*_ receives all signals produced by cell type *C*_*j*_ if *A*(*j*,*i*)=1, where *A*(*j*,*i*)=Ber(*P*_adj_). *C*_*i*_ receives no signals from cell type *C*_*j*_ if *A*(*j*,*i*)=0 (Fig. 1d). Cells can only receive signals from other cell types present in the same time step. Let $T_{t} = \{0,1\}^{2^{N}}$ be a binary vector, where *T*_*t*_(*i*)=1 if cell type *C*_*i*_ is present in the time step *t*. *T*_*t*_ represents the state of the organism at time step *t*. For some cell type, *C*_*i*_ present at time step *t*, let ${C^{\text {sig}}_{i}}$ represent its state immediately after signal exchange. In cell types that receive a signal, the corresponding genes are set to 1 (Fig. 1f). That is:

$$\begin{array}{*{20}l} &{} {C^{\text{sig}}_{i}}(k) = 1,\\ &{}\text{if}\,\, (C_{i}(k)=1) \vee \left({\sum\nolimits}_{j=1}^{2^{N}}(A(j,i) \times {\text{SG}}_{j}(k) \times T_{t}(j)) > 0\right) \end{array} $$

#### Gene regulation

We define gene regulation in the model as a set of stable cell types and cell types in the basins of these stable cell types. As mentioned earlier, stable cell types need not be fixed points (single-cell state) of the gene regulatory network; they can also be an oscillation (multiple cell states). Oscillatory stable cell types are represented as the set of all cell states that compose the oscillation.

Formally, a system with *N* genes can have *n*≤2^*N*^ stable cell types {*S*_1_,*S*_2_,…,*S*_*n*_}, where *S*_*x*_ is itself a collection of *n*_*x*_ cell states $\left \{C_{x_{1}}, \ldots C_{x_{n_{x}}}\right \}$ such that $\phantom {\dot {i}\!}x_{1} < x_{2} <\ldots < x_{n_{x}}$. For any two cell types *S*_*x*_ and *S*_*y*_, if *x*<*y*, then *x*_1_<*y*_1_.

We encode gene regulation in a binary matrix $\phantom {\dot {i}\!}GR_{2^{N}\times 2^{N}}$. To generate *GR* for a given organism, we pick the number of stable cell types *n*≤2^*N*^ according to the uniform random distribution. First, we assign cell states that form the basins of these stable cell types: Cell states are uniform randomly partitioned among the *n* basins. We then choose cell states that form the stable cell type from within the corresponding basins: let *B*_*x*_ be a basin, then for some *j* such that (*C*_*j*_∈*B*_*x*_),(*C*_*j*_∈*S*_*x*_) with probability 0.5. For all *i* such that *C*_*i*_∈*B*_*x*_,GR(*i*,*j*)=1 if (*C*_*j*_∈*S*_*x*_).

#### Homeostatic organisms and their cell type lineage graphs

Let us consider an organism in state *T*_*t*_ at time step *t*. Right after cell division, let the state of the organism be represented by ${T^{\text {div}}_{t}}$. After division, the organism is composed of all the daughter cells produced in that time step. That is:

$${T^{\text{div}}_{t}}(i) = 1,\,\, \text{if}\,\, \exists j \leq 2^{N}\,\, \text{s.t.}\,\, \left(T_{t}(j) = 1\right) \wedge ({\text{CD}}(j,i) = 1) $$

These daughter cells exchange signals among themselves. Let ${T^{\text {sig}}_{t}}$ represent the state of the organism right after signal exchange. Then:

$$\begin{array}{@{}rcl@{}} {T^{\text{sig}}_{t}}(i) &=& 1,\,\, \text{if}\,\, \exists j1 \leq 2^{N}\,\,\text{s.t.}\,\, {T^{\text{div}}_{t}}(j1) = 1,\,\, \text{where}\\ && \forall k\,\,\text{s.t.}\,\, C_{i}(k) = 0, C_{j1}(k) = 0,\,\, \text{and}\\ \forall k\, \text{s.t.}\, C_{i}(k)\!\! &=& \!\!1, \left(C_{j1}(k) = 1\right)\\&&\!\vee\!\left(\!{\sum\nolimits}^{2^{N}}_{j2=1}\!(A(j2,j1) \!\times\! {\text{SG}}_{j2}(k) \!\times\! {T^{\text{div}}_{t}}(j2))\!>\!0\!\right) \end{array} $$

The signals received by a cell type activates its gene regulatory network. Gene regulation updates the set of cell types according to the following expression: ∀*i*≤2^*N*^,

$$T_{t+1}(i) = 1,\,\, \text{if}\,\, \exists j\,\, \text{s.t.}\,\, \left({T^{\text{sig}}_{t}}(j)=1\right) \wedge ({\text{GR}}(j,i)=1) $$

Therefore, the organism is only composed of stable cell types. Let the system have *n*≤2^*N*^ stable cell states. Then, we can equivalently represent the state of the organism at time step *t* as a binary vector $T^{SC}_{t} = [0,1]^{n}$, such that for *x*∈{1,2,…*n*}.

$$T^{SC}_{t}(x) = 1 \iff \left(T_{t}(i) = 1\right) \wedge \left(\exists C_{i} \in S_{x}\right) $$

We call states of the organism such that $T^{SC}_{t+1} = T^{SC}_{t}$ homeostatic organisms (Fig. 1f, g).

We represent the homeostatic organism as a cell type lineage graph. The nodes of the graph represent stable cell states that are present in the homeostatic organism, and directed edges represent lineage relationships between these stable cell states. Let the stable cell states *S*_*x*1_ and *S*_*x*2_ both be present in the final organism, and let them be represented by nodes *V*_*a*_ and *V*_*b*_ of the lineage graph, respectively. Then, there is an edge from *V*_*a*_ to *V*_*b*_ if one of the daughter cells of *S*_*x*1_ gives rise to *S*_*x*2_ after one round of cell signaling and gene regulation (Fig. 1g). That is:

$$\begin{aligned} &&\text{Let} C_{i} \in S_{x1}\,\, \text{and}\,\, C_{l} \in S_{x2}.\\ &&\text{Then, there is an edge}\,\, V_{a} \rightarrow V_{b} \,\,\,\text{if}\\ && \exists j\,\, \text{s.t.}\,\, {\text{CD}}(i,j)=1\\ &&\text{and, in this organism}, {C^{\text{sig}}_{j}} = C_{k} \\ &&\text{where}\,\, {\text{GR}}(k,l) = 1 \end{aligned} $$

### Assignment of topologies to lineage graphs

We categorize lineage graphs into 6 topologies: unicellular, strongly connected component(SCC), cyclic, chain, tree, and other directed acyclic graphs (DAG). We ignore self-edges while assigning these topologies. A lineage graph is called *unicellular* if it has only a single node. For all other topologies, we used the networkx (version 2.2) module of Python3.6. A lineage graph is called *SCC* if the graph has more than 1 node and contains a single strongly connected component, it is called *cyclic* if the graph contains cycles and has more than one strongly connected component, it is called a *chain* if networkx classifies it as a tree and the maximum in-degree and out-degree are 1, it is called a *tree* if networkx classifies it as a tree and maximum in-dergee or out-degree is greater than 1, and it is called a *DAG* if networkx classifies it as a directed acyclic graph but not a tree.

### Lineage graph randomization protocol

We represent a lineage graph with *e* edges as a matrix *E*_*e*×2_, where *E*(*i*,1) and *E*(*i*,2) represent the source and the target node of edge *i*, respectively. To randomize lineage graphs, we used a protocol that preserves in- and out-degrees of each node; we randomly choose pairs of edges from the graph and swap their target nodes. Let the randomized graph *E*_rand_ be initially identical to *E*. Then, for any two edges of the lineage graph *i*,*j*, we propose a swap:

$${E_{\text{rand}}}(i,2) = {E_{\text{rand}}}(j,2),\,\, \text{and}\,\, {E_{\text{rand}}}(j,2) = {E_{\text{rand}}}(i,2) $$

The swap is accepted if there is no edge *k* such that:

$$\begin{array}{@{}rcl@{}} &&({E_{\text{rand}}}(k,1) = {E_{\text{rand}}}(i,1))\wedge({E_{\text{rand}}}(k,2) = {E_{\text{rand}}}(j,2)), \text{or}\\&& ({E_{\text{rand}}}(k,1) = {E_{\text{rand}}}(j,1))\wedge({E_{\text{rand}}}(k,2) = {E_{\text{rand}}}(i,2)) \end{array} $$

The above condition ensures that the total number of unique edges in *E* and *E*_rand_ remain the same. We swap edges 1000 times for each lineage graph to randomize it.

### Independent and intrinsically independent cell types

We call a cell type *independent* if it has the same cell fate when grown outside the organism as it does when it is a part of the organism. The cell fate ${C^{\text {fate}}_{i}}$ of some cell type *C*_*i*_ in the organism is given by the set of cell types receiving an edge from the node *C*_*i*_ in the organism’s lineage graph. To decide whether a given cell type *C*_*i*_ is independent or not, we separate this cell type from the rest of the organism and allow it to undergo one round of cell division, signaling, and gene regulation, according to the same matrices CD,SG, *A*, and GR that were used to generate the organism from which it was taken. Let us call the resulting set of cell types ${C^{\text {reg}}_{i}}$. We call the cell *C*_*i*_ independent if ${C^{\text {reg}}_{i}}$ is identical to ${C^{\text {fate}}_{i}}$.

For some cell types, the basis of their independence is an insensitivity to signals produced in the organism. In such a case, the set of signals produced by the daughter cells of the cell type is sufficient to satisfy the maximum set of signals that each of the daughter cells can receive.

Let the set of daughter cells of cell type *C*_*i*_ in an organism be *D*_*i*_. ∀*C*_*j*_∈*D*_*i*_ let ${\text {Rec}^{\text {all}}_{j}}$ represent the maximal set of signals that it can receive, when all 2^*N*^ possible cell types are present together. That is, for all signaling molecules *k* such that SG(*k*)=1,

$$ {\text{Rec}^{\text{all}}_{j}}(k) = 1,\,\, \text{if}\,\, \Sigma^{2^{N}}_{l=1}\left(A(l,j) \wedge (C_{l}(k) = 1)\right) $$

And let ${\text {Rec}}^{D}_{j}$ be the set of signals it receives from within the set of cells *D*_*i*_, i.e.,

$${\text{Rec}}^{D}_{j}(k) = 1,\,\,\text{if}\,\, \Sigma_{C_{l} \in D_{i}} \left(A(l,j) \wedge (C_{l}(k) = 1)\right) $$

If for all $C_{j} \in D_{i},{\text {Rec}^{\text {all}}_{j}}$ = ${\text {Rec}}^{D}_{j},C_{i}$ is *intrinsically independent*.

## Supplementary Information

**Additional file 1** Figures S1–S19. **Figure S1**- Modified segment polarity network. **Figure S2**- Developmental rules matrices for the modified segment polarity network. **Figure S3**- Distribution of basin sizes of lineage graphs. **Figure S4**- Effect of parameters on lineage graph sizes. **Figure S5**- Lineage graphs versus Erdos-Renyi random graphs. **Figure S6**- Lineage graph topologies. **Figure S7**- Effect of genome regulation. **Figure S8**- Distribution of topologies of randomized graphs. **Figure S9**- Graph size distributions for different topologies. **Figure S10**- Properties of tree-type graphs. **Figure S11**- Properties of DAG-type graphs. **Figure S12**- Regenerative capacity and isomorphic graphs. **Figure S13**- Box plots for regenerative capacity of lineage graphs. **Figure S14**- Stacked histograms showing intrinsic independence of types. **Figure S15**- Independent pluripotent cell-types. **Figure S16**- Comparison of parameters that generate regenerative acyclic lineage graphs with pluripotent root nodes versus those with non-root node pluripotent cells. **Figure S17**- Effect of including cell-death in the model. **Figure S18**- Properties of ’acyclized’ cyclic graphs. **Figure S19**- Box plots for regenerative capacities using relaxed definitions for acyclic graphs and trees.

## Data Availability

Octave5.2.0 and Python3.6 code were used in this work. All data generated or analyzed during this study are included in this published article and its supplementary information files. All data and codes used to generate and analyze the data are available in the BioModels repository [42] and assigned the identifier MODEL2103180001 [43]. Declarations

## Abbreviations

DAG: Directed acyclic graph
RBN: Random Boolean network
ER: Erdos-Renyi
SCC: Strongly connected component
WBR: Whole-body regeneration

## References

[1] Milo R, Jorgensen P, Moran U, Weber G, Springer M (2009). Bionumbers — the database of key numbers in molecular and cell biology. Nucleic Acids Res.

[2] Hwang JS, Ohyanagi H, Hayakawa S, Osato N, Nishimiya-Fujisawa C, Ikeo K, David CN, Fujisawa T, Gojobori T (2007). The evolutionary emergence of cell type-specific genes inferred from the gene expression analysis of hydra. Proc Natl Acad Sci.

[3] Meyerowitz EM (2002). Plants compared to animals: the broadest comparative study of development. Science.

[4] Von Dassow G, Meir E, Munro EM, Odell GM (2000). The segment polarity network is a robust developmental module. Nature.

[5] Volkening A, Sandstede B (2015). Modelling stripe formation in zebrafish: an agent-based approach. J R Soc Interface.

[6] Ben-Zvi D, Fainsod A, Shilo B-Z, Barkai N (2014). Scaling of dorsal-ventral patterning in the xenopus laevis embryo. Bioessays.

[7] Kester L, van Oudenaarden A (2018). Single-cell transcriptomics meets lineage tracing. Cell Stem Cell.

[8] Bolker JA (2000). Modularity in development and why it matters to evo-devo. Am Zool.

[9] Albert R, Othmer HG (2003). The topology of the regulatory interactions predicts the expression pattern of the segment polarity genes in drosophila melanogaster. J Theor Biol.

[10] Garfield DA, Runcie DE, Babbitt CC, Haygood R, Nielsen WJ, Wray GA (2013). The impact of gene expression variation on the robustness and evolvability of a developmental gene regulatory network. PLoS Biol.

[11] Alberts B, Johnson A, Lewis J, Raff M, Roberts K, Walter P (2002). Universal mechanisms of animal development. Molecular Biology of the Cell.

[12] Gilbert SF, Barresi M (2017). Developmental biology, 2016. Am J Med Genet A.

[13] Sharpe J (2017). Computer modeling in developmental biology: growing today, essential tomorrow. Development.

[14] Goss RJ (1992). The evolution of regeneration: adaptive or inherent?. J Theor Biol.

[15] Reddien PW (2018). The cellular and molecular basis for planarian regeneration. Cell.

[16] Brown FD, Swalla BJ (2012). Evolution and development of budding by stem cells: ascidian coloniality as a case study. Dev Biol.

[17] Mochizuki A, Fiedler B, Kurosawa G, Saito D (2013). Dynamics and control at feedback vertex sets. ii: A faithful monitor to determine the diversity of molecular activities in regulatory networks. J Theor Biol.

[18] Knoblich JA (2008). Mechanisms of asymmetric stem cell division. Cell.

[19] Altschuler SJ, Wu LF (2010). Cellular heterogeneity: do differences make a difference?. Cell.

[20] Klein AM, Simons BD (2011). Universal patterns of stem cell fate in cycling adult tissues. Development.

[21] Kawaguchi D, Furutachi S, Kawai H, Hozumi K, Gotoh Y (2013). Dll1 maintains quiescence of adult neural stem cells and segregates asymmetrically during mitosis. Nat Commun.

[22] Gershenson C. Introduction to random boolean networks. 2004. http://arxiv.org/abs/nlin/0408006.

[23] Xia B, Yanai I (2019). A periodic table of cell types. Development.

[24] Graham LK, Wilcox LW (2000). The origin of alternation of generations in land plants: a focus on matrotrophy and hexose transport. Philos Trans R Soc Lond B Biol Sci.

[25] Matt G, Umen J (2016). Volvox: a simple algal model for embryogenesis, morphogenesis and cellular differentiation. Dev Biol.

[26] Pellin D, Loperfido M, Baricordi C, Wolock SL, Montepeloso A, Weinberg OK, Biffi A, Klein AM, Biasco L (2019). A comprehensive single cell transcriptional landscape of human hematopoietic progenitors. Nat Commun.

[27] Siebert S, Farrell JA, Cazet JF, Abeykoon Y, Primack AS, Schnitzler CE, Juliano CE (2019). Stem cell differentiation trajectories in hydra resolved at single-cell resolution. Science.

[28] Gehart H, van Es JH, Hamer K, Beumer J, Kretzschmar K, Dekkers JF, Rios A, Clevers H (2019). Identification of enteroendocrine regulators by real-time single-cell differentiation mapping. Cell.

[29] Tritschler S, Büttner M, Fischer DS, Lange M, Bergen V, Lickert H, Theis FJ (2019). Concepts and limitations for learning developmental trajectories from single cell genomics. Development.

[30] Saelens W, Cannoodt R, Todorov H, Saeys Y (2019). A comparison of single-cell trajectory inference methods. Nat Biotechnol.

[31] Nishida H (2005). Specification of embryonic axis and mosaic development in ascidians. Dev Dyn Off Publ Am Assoc Anatomists.

[32] Alié A, Hiebert LS, Scelzo M, Tiozzo S (2021). The eventful history of nonembryonic development in tunicates. J Exp Zool (Mol Dev Evol).

[33] Menchero S, Rollan I, Lopez-Izquierdo A, Andreu MJ, De Aja JS, Kang M, Adan J, Benedito R, Rayon T, Hadjantonakis A-K (2019). Transitions in cell potency during early mouse development are driven by Notch. Elife.

[34] Chaves M, Albert R, Sontag ED (2005). Robustness and fragility of Boolean models for genetic regulatory networks. J Theor Biol.

[35] Cheng X, Sun M, Socolar JE (2013). Autonomous Boolean modelling of developmental gene regulatory networks. J R Soc Interface.

[36] Zattara EE, Fernández-Álvarez FA, Hiebert TC, Bely AE, Norenburg JL (2019). A phylum-wide survey reveals multiple independent gains of head regeneration in nemertea. Proc R Soc B.

[37] Claessen D, Rozen DE, Kuipers OP, Søgaard-Andersen L, Van Wezel GP (2014). Bacterial solutions to multicellularity: a tale of biofilms, filaments and fruiting bodies. Nat Rev Microbiol.

[38] Bely AE (2010). Evolutionary loss of animal regeneration: pattern and process. Integr Comp Biol.

[39] Plass M, Solana J, Wolf FA, Ayoub S, Misios A, Glažar P, Obermayer B, Theis FJ, Kocks C, Rajewsky N (2018). Cell type atlas and lineage tree of a whole complex animal by single-cell transcriptomics. Science.

[40] Wagner DE, Weinreb C, Collins ZM, Briggs JA, Megason SG, Klein AM (2018). Single-cell mapping of gene expression landscapes and lineage in the zebrafish embryo. Science.

[41] Lei K, McKinney SA, Ross EJ, Lee H-C, Alvarado AS. Cultured pluripotent planarian stem cells retain potency and express proteins from exogenously introduced mrnas. BioRxiv. 2019:573725.

[42] Malik-Sheriff RS, Glont M, Nguyen TVN, Tiwari K, Roberts MG, Xavier A, Vu MT, Men J, Maire M, Kananathan S, Fairbanks EL, Meyer JP, Arankalle C, Varusai TM, Knight-Schrijver V, Li L, Dueñas-Roca C, Dass G, Keating SM, Park YM, Buso N, Rodriguez N, Hucka M, Hermjakob H (2020). BioModels — 15 years of sharing computational models in life science. Nucleic Acids Res.

[43] Mani S, Tlusty T. ManiTlusty2021 - generative development model. BioModels.MODEL2103180001. 2021. https://www.ebi.ac.uk/biomodels/MODEL2103180001.

