# Supplementary material for: A comprehensive survey of developmental programs reveals a dearth of tree-like lineage graphs and ubiquitous regeneration
Source: BMC Biol. 2021 May 21;19:111. doi: 10.1186/s12915-021-01013-4 (PMC8140435; doi:10.1186/s12915-021-01013-4)
Supplement: Supplementary file 1 — Additional file 1 Figures S1–S19. Figure S1- Modified segment polarity network. Figure S2- Developmental rules matrices for the modified segment polarity network. Figure S3- Distribution of basin sizes of lineage graphs. Figure S4- Effect of parameters on lineage graph sizes. Figure S5- Lineage graphs versus Erdos-Renyi random graphs. Figure S6- Lineage graph topologies. Figure S7- Effect of genome regulation. Figure S8- Distribution of topologies of randomized graphs. Figure S9- Graph size distributions for different topologies. Figure S10- Properties of tree-type graphs. Figure S11- Properties of DAG-type graphs. Figure S12- Regenerative capacity and isomorphic graphs. Figure S13- Box plots for regenerative capacity of lineage graphs. Figure S14- Stacked histograms showing intrinsic independence of types. Figure S15- Independent pluripotent cell-types. Figure S16- Comparison of parameters that generate regenerative acyclic lineage graphs with pluripotent root nodes versus those with non-root node pluripotent cells. Figure S17- Effect of including cell-death in the model. Figure S18- Properties of ’acyclized’ cyclic graphs. Figure S19- Box plots for regenerative capacities using relaxed definitions for acyclic graphs and trees. [file 12915_2021_1013_MOESM1_ESM.pdf]

A comprehensive survey of developmental programs reveals ubiquitous regeneration and dearth of tree-like lineage graphs

Somya Mani [1,*] and Tsvi Tlusty [1,2,3,*]

[1]Institute for Basic Science – Center for Soft and Living Matter, Ulsan-44919, South Korea
[2]Department of Physics, Ulsan National Institute of Science and Technology (UNIST), Ulsan 44919, Korea
[3]Department of Chemistry, Ulsan National Institute of Science and Technology (UNIST), Ulsan 44919, Korea
[*]Correspondence: somyamn@gmail.com (S.M.), tsvitlusty@gmail.com (T.T.)

March 22, 2021

1 Supplementary material

1.1 Drosophila segement polarity network expressed in terms of the generative model

In Drosophila embryos, segment polarity genes maintain borders of parasegments, which are 4 cells wide. Within each parasegment, the polarity genes are expressed in characteristic stripes. In Albert and Othmer (2003), the authors demonstrated that the gene regulatory network responsible for the pattern of gene expression in this system can be modeled as a Boolean logical network. In the following, we examine the Drosophila segment polarity network in terms of our generative model.

The network consists of 15 nodes: en, wg, hh, ptc and ci represent mRNAs, and SLP, EN, WG, HH, PTC, SMO, PH, CI, CIA and CIR represent proteins. Of these, WG, hh and HH act as signals. Signaling molecules HH and WG do not participate in regulation within the cells that produce them, rather they act only in cells that receive them as signals. In order to incorporate this feature, we represent each cell in the parasegment as two model cells; production of all non-signal molecules takes place in one of the cells, and molecules responsible for regulation of signal molecule production are exported to the second cell, from which signal molecules are secreted (Fig.S1(A,B)). In this sense, the second cell acts as a special compartment which insulates the gene network in the first cell from regulation by signal molecules produced within the same cell.

In this system, signals are exchanged only between neighbouring cells. Accordingly, in our model, cell positions can be expressed as additional 'genes', whose states do not change. For example, to express the positions of the 4×2 cells in this system, we use 3 additional 'genes' (Fig.S1(C)). In this system, signal exchange only depends on these 'positional genes', and does not depend on the states of the other genes.

Thus, our model is capable of expressing spatial arrangement of cells, and complex signaling mechanisms, although, it comes at the cost of an increase in system size. In Fig. S2, we show the signaling vector SG, and portions of the cellular adjacency matrix A, and gene regulation matrix GR relevant to the steady state of the wild-type segment polarity network. The authors assume symmetric cell-division in Albert and Othmer (2003), and we do the same; therefore we do not show the cell-division matrix CD here.

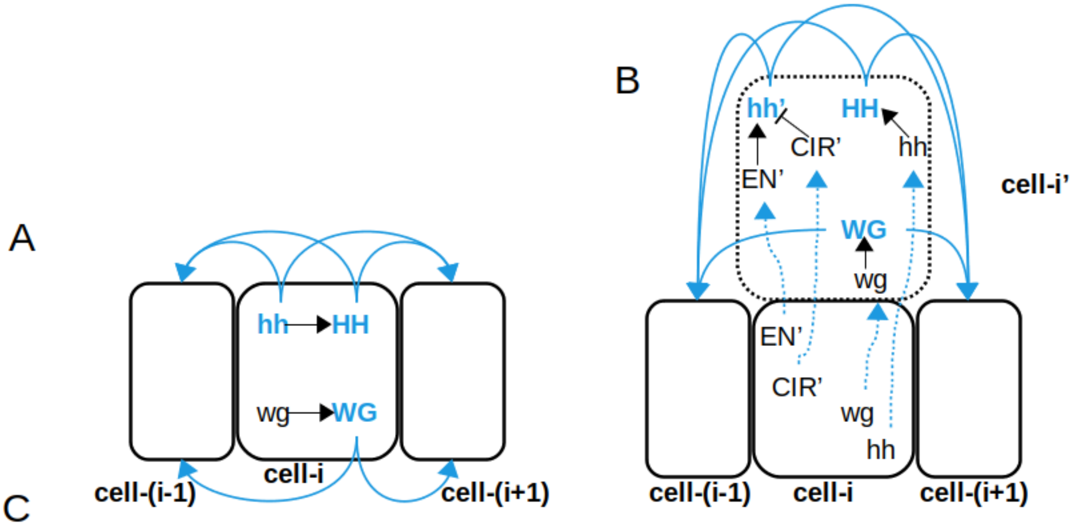

C

Cell index	SLP	wg	WG	en	EN	hh	HH	ptc	PTC	PH	SMO	ci	CI	CIA	CIR	hh'	EN'	CIR'	Bits for cell position			
459792	0	0	0	1	1	1	0	0	0	0	1	0	0	0	0	0	1	0	0	0	0	cell-1
32801	0	0	0	0	0	0	1	0	0	0	0	0	0	0	0	1	0	0	0	0	1	cell-1'
32642	0	0	0	0	0	0	0	1	1	1	1	1	1	1	0	0	0	0	0	1	0	cell-2
3	0	0	0	0	0	0	0	0	0	0	0	0	0	0	0	0	0	0	0	1	1	cell-2'
2102092	1	0	0	0	0	0	0	0	1	0	0	1	1	0	1	0	0	1	1	0	0	cell-3
5	0	0	0	0	0	0	0	0	0	0	0	0	0	0	0	0	0	0	1	0	1	cell-3'
3178374	1	1	0	0	0	0	0	1	1	1	1	1	1	1	0	0	0	0	1	1	0	cell-4
524295	0	0	1	0	0	0	0	0	0	0	0	0	0	0	0	0	0	0	1	1	1	cell-4'

Figure S1: **Modified segment polarity network (A)** Signaling in the original model, as in Albert and Othmer (2003)). Signaling molecules are labeled in blue. Blue edges represent signal transduction, and black edges represent 'gene'-regulation. **(B)** Modified structure of segment polarity network. We introduce a new cell, shown here with a dotted outline, adjacent to the original cell, which acts like an insulated compartment of this cell. All signals are transmitted via this new cell to neighbouring cells. **(C)** Steady state of the modified network that corresponds to wild-type stripe pattern in Drosophila, as reported in Albert and Othmer (2003). Each row is a cell-type, and columns represent states of 'genes'. 1 implies presence of the gene product, and 0 implies absence of the gene product. There are 21 genes in the system: the first 15 genes are the original mRNAs and proteins used to construct the regulatory network in Albert and Othmer (2003), and the next 3 genes represent 'mirrors' of hh, EN and CIR which are used for signaling purposes. The last 3 'genes' encode the position of the cell along the anterio-posterior axis; cell-1 is the most anterior and cell-4 is the most posterior. Cells 1'-4' represent the new cells we introduce for signal transduction. Each cell is indexed by the decimal number obtained upon converting the corresponding 21-length binary vector.

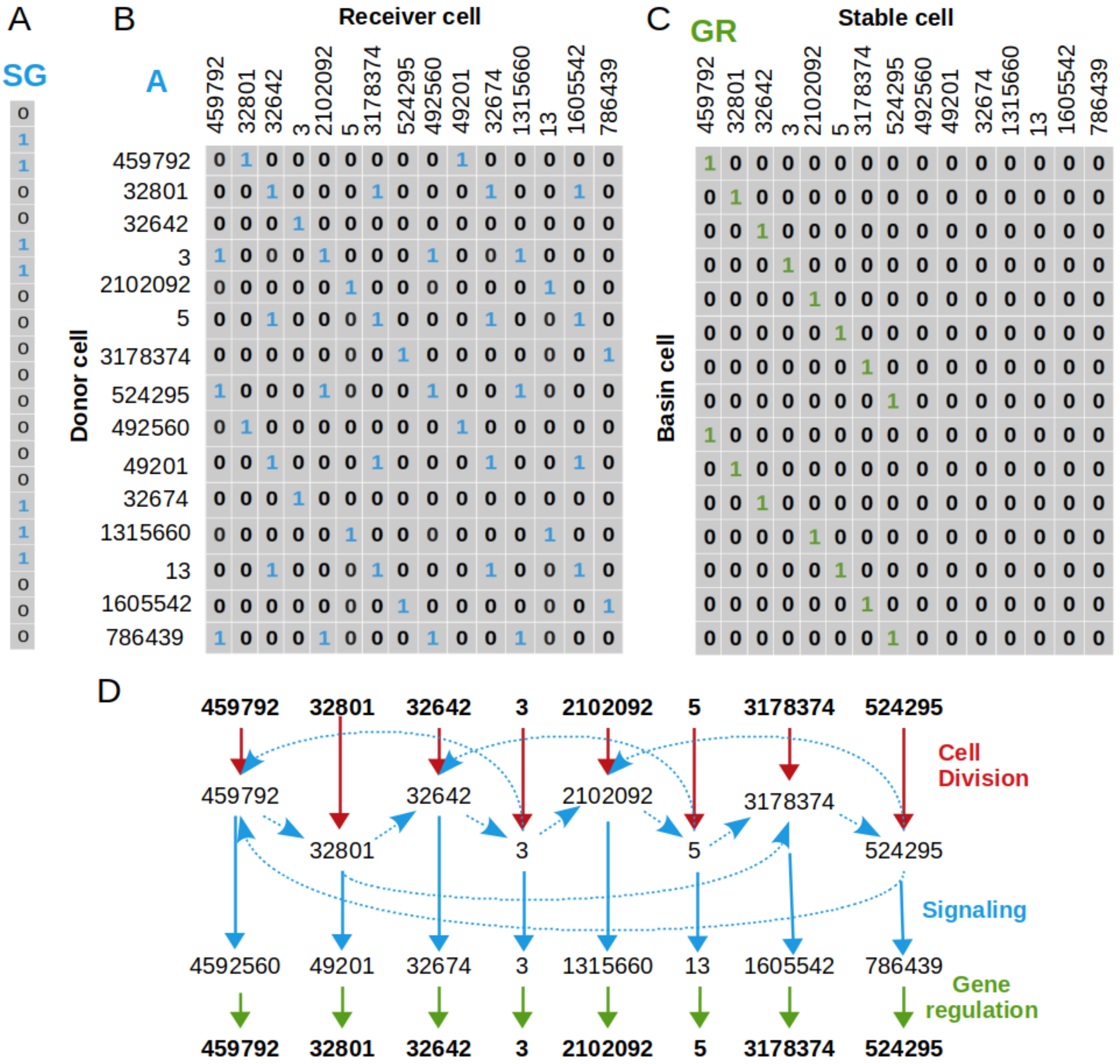

Figure S2: **Developmental rules matrices for the modified segment polarity network (A)** signaling vector SG, **(B)** Cellular adjacency matrix A. Note that cellular adjacency is completely determined by cell positions; Cells-1-4 only pass on molecules to cells-1'-4' respectively, and a cell-i' only passes on signals to cell-(i-1) and cell-(i+1). Periodic boundary conditions are employed here, which implies that cell-1 and cell-4 are neighbours. **(C)** Gene regulation matrix GR. The first 8 cell-types correspond to the stable state, as in Fig.S1(C). In **B** and **C**, only the relevant parts of the rules matrices are shown. The full matrices are of size $2^{21} \times 2^{21}$. **(D)** Schematic diagram of signaling and gene regulation in determining the wild-type steady state of the *Drosophila* segment polarity network. Numbers represent the indices of different cell-types. Red arrows represent cell-division, which is symmetric in this case. Dashed blue arrows represent signal exchange among cell-types and solid blue arrows represent changes in cell-types due to signal exchange. Green arrows represent gene regulation.

1.2 basin sizes of gene regulatory networks

In the model, stable cell-types and cells in their basins of attraction are randomly chosen. In gene regulatory networks with N genes, between $50\% - 75\%$ of 2^N cells are stable cell-types (Fig.S3(B)). On average, basins of these stable cell-types are small, and most basins contain a single cell-type (Fig.S3(B,C)).

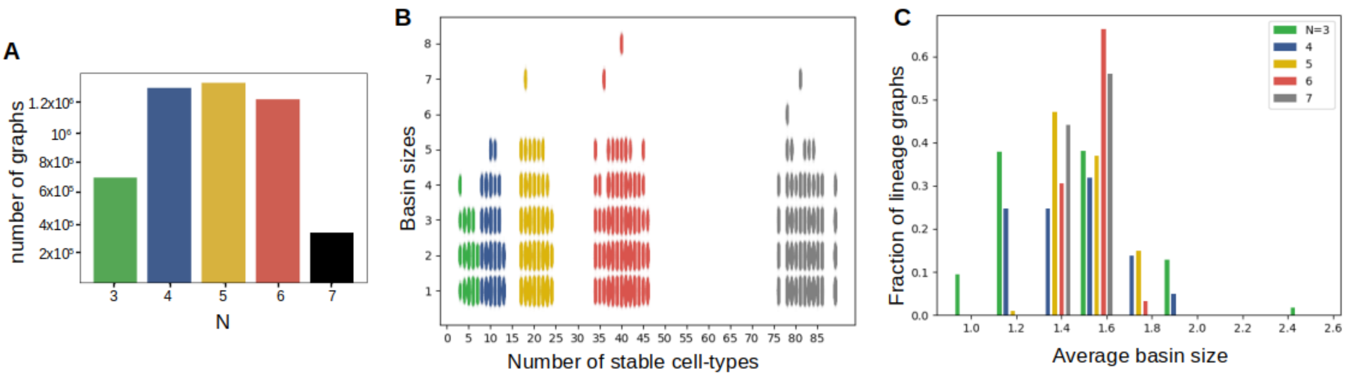

Figure S3: **Distribution of basin sizes of lineage graphs.** 4852994 graphs were used to generate these plots. **(A)** Number of model generated homeostatic organisms in the data at different values of N. **(B)** Basin sizes in organisms containing different numbers of stable cell-types. (**C**) Histogram of average basin size for all homeostatic organisms.

1.3 Lineage graph size

While a majority of graphs in our data are small (1-5 nodes), the largest graphs have 89 nodes (Fig.S4(A)). The number of nodes in lineage graphs follows closely the diversity of daughter cell-types produced (Fig.S4(C,D)). At very low P_{asym}, cells produce daughters cells identical to themselves, and at very high P_{asym}, most daughter cells are of the type $[0, 0, ...0]$. Therefore at these values, diversity of daughter cells, and correspondingly the number of nodes in lineage graphs, is low. At other values of P_{asym}, the number of nodes stays level and decreases slowly beyond $P_{\text{asym}} = 0.5$ (Fig.S4(C)). Number of nodes decreases as P_{sig} increases (Fig.S4(D)). Intuitively, high levels of signaling causes genes in a 1 state to 'spread out', effectively leading to a homogenization of cell-types. The sharp decrease in the number of nodes in response to increase in P_{adj} indicates that a low level of inter-cellular connectivity is sufficient for signals to percolate throughout the organism (Fig.S4(E), Fig.S5(B)).

We compared the properties of lineage graphs in our data with those expected for Erdos-Renyi random graphs (ER graphs) of similar size. In ER graphs,there is a fixed probability, p of any two nodes in the graph being connected by an edge (Erdös and Rényi, 1959). Therefore, on average, the number of edges in a graph with n nodes is proportional to n^2. Increasing the number of nodes to $c * n$ increases the number of edges to $c^2 * n^2$. We determined the number of edges in lineage graphs with $n = [1, 2, 3, 4, 5, 6, 7, 8, 9, 10]$ nodes and calculated the number of edges expected in graphs with twice the number of nodes if these were ER graphs. We then compared the number of edges in our data with $n = [2, 4, 6, 8, 10, 12, 14, 16, 18, 20]$ nodes with what we would expect for ER graphs. Compared to ER graphs, the rate of growth of number of edges in lineage graphs in our data is noticeably slower (Fig.S5(A)).

The number of nodes in lineage graphs decreases sharply with the parameter P_{adj} (Fig.S4(E)). We show here that this occurs because even at low values of P_{adj}, cell-types in organisms are connected enough that the fraction of cell-types receiving all signals produced in the organisms reaches a maximum (Fig.S5(B)).

The effect of the parameter P_{asym} on the number of nodes in lineage graphs can be explained in terms of its effect on the number of distinct daughter cell-types produced (Fig.S5(C)). The number of distinct daughter cells produced at different values of P_{asym} is related to the average fraction of genes in a 1 state in these daughter cells. Among all possible cell-types with N genes, most cell-types tend to have about half their genes in a 1 state, and very few cell-types contain fewer, or more genes in a 1 state (Fig.S5(D:inset)). Therefore, when $0.2 < P_{\text{asym}} < 0.6$, where on average, daughter cells have about half their genes in a 1 state,

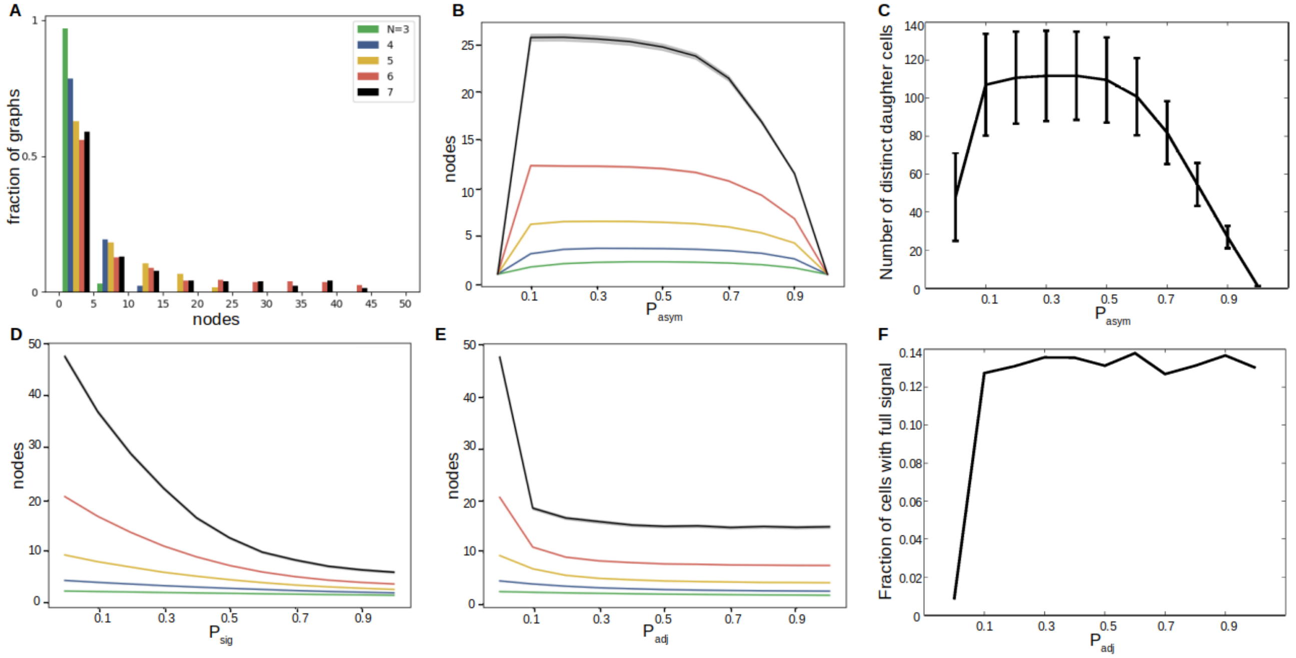

Figure S4: **Effect of parameters on lineage graph sizes.** **(A)** Histogram of number of nodes in lineage graphs obtained with different N. Histogram bins are of size 5. **(B)** Number of nodes in lineage graphs obtained at different N as a function of P_{asym}. **(C)** Average number of distinct daughter cells produced in an organism as a function of P_{asym}. At each value of P_{asym}, 10,000 'organisms' with $N = 7$ genes, composed of randomly chosen cell-types were used to generate these graphs. Error-bars indicate standard deviation. **(D)** Number of nodes in lineage graphs obtained at different N as a function of P_{sig}. **(E)** Number of nodes in lineage graphs obtained at different N as a function of P_{adj}. **(F)** Effect of P_{adj} on signal reception. At each value of P_{adj}, 1000 random signaling vectors SG for $N = 7$ organisms, generated at $P_{\mathrm{sig}} = 0.5$ were used. In each organism, the set of signals received by a randomly chosen cell-type, from all 2^N possible cell-types in the system was assessed. The horizontal axis represents P_{adj}, and the vertical axis represents the fraction of cell-types out of 1000, that received all possible signals. In **(B,D,E)** thick lines represent the mean and shaded regions around the lines represent standard deviation (the shaded regions are hard to see because the standard deviations are low). 4852994 graphs were used to generate plots in **(A,B,D,E)**.

organisms produce the most number of distinct daughter cells, and at $P_{\mathrm{asym}} < 0.2$ and $P_{\mathrm{asym}} > 0.6$, fewer distinct daughter cells are produced (Fig.S5(D)).

1.4 Lineage graph topologies and graph randomization

Lineage graphs can be either cyclic or acyclic. The acyclic graphs can be further classified into (i) chains, (ii) trees (acyclic graphs with branches) and (iii) DAGs (Directed Acyclic Graphs, which contain edges connecting different branches). And cyclic lineage graphs can be further classified into (i) unicellular (single cell-type), (ii) SCC (Strongly Connected Component – all paths are cyclic), and (iii) cyclic (contains both cyclic and acyclic paths), (iv) chains (acyclic graphs with no branches) (Fig.S6(A)).

Although all topologies are spread widely across parameter space, different topologies are enriched in different regions of parameter space (Fig.S6(B,C)). No parameter region is monopolized by a single topology, except at extreme values of P_{asym}, where, as discussed earlier, most graphs are unicellular.

In our data, the most sparsely sampled ingredient is genome regulation. Thus, we wanted to find out the extent to which graph topology distributions (i.e. fractions of all graphs which are unicellular, cyclic/SCC, chains, trees or DAGs) depend on the details of genome regulation matrices we use. To do this, we compare lineage graph topology distributions produced by bootstrap samples to those in the full data. The topology distributions were obtained by pooling all graphs produced in the sample across

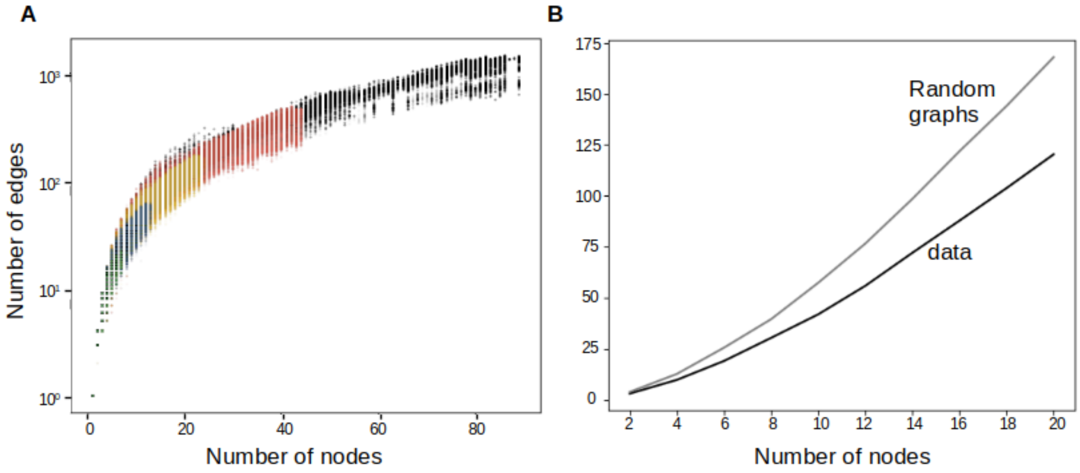

Figure S5: **Lineage graphs versus Erdos-Renyi random graphs. (A)** scatter plot of number of edges and number of nodes in lineage graphs. Transparency has been added to points to make density of points more apparent. 4852994 graphs were used to generate this plot.**(B)** A comparison of growth rate of the number of edges with number of nodes in lineage graphs of our data, versus that expected of Erdos-Renyi random graphs. 4532110 graphs were used to generate this plot.

all parameters. The analysis indicates that graph topology distributions do not depend strongly on the details of the genome, and even bootstrap samples one-fourth the size of the original data produce similar topology distributions (Fig. S7).

To a large extent, lineage graph topologies can be characterized by their in-degree and out-degree distributions. For instance, in chains, in-degrees and out-degrees are at most 1, whereas in SCCs, in-degrees and out-degrees are at least 1 (Fig.S8(A)). Therefore, for the most part, we can explain the model's propensity to generate certain topologies, in terms of its propensity to generate certain in-degree and out-degree distributions.

We randomized lineage graphs generated with our model while keeping node in-degrees and out-degrees unchanged. Topology distribution largely remains unchanged upon randomization (Fig.S8(C,D)), and not many graphs change their topology upon randomization (Fig.S8(B)). Although, we find that the proportion of acyclic graphs decreases slightly, from 24% in model generated graphs, to 19% in randomized graphs.

1.5 Characteristics of lineage graphs with different topologies: graph size

Different graph topologies are different in their graph size distributions. While SCC and cyclic graphs span a large range of graph sizes (Fig.S9(A,B)), trees and chains tend to be notably small (Fig.S9(C,E)). DAG type graphs can have moderately large number of nodes (Fig.S9(D)).

1.6 Characteristics of tree-type lineage graphs

Tree-type graphs can be further characterized as divergent or convergent trees. We call graph nodes with in-degrees > 1 convergent, and nodes with out-degrees > 1 divergent. Note that by this definition, the same node is allowed to be both convergent and divergent. For some tree-like graph with n nodes and n_e edges, let in_i and out_i be the in-degree and out-degree of the ith node respectively. We define for this graph a number g_c as the sum of in-degrees of all convergent nodes, and a number g_d as the sum of out-degrees of all divergent nodes, i.e.;

$$g_c = \Sigma in_i, \forall i \text{ s.t. } in_i > 1,$$

$$g_d = \Sigma out_i, \forall i \text{ s.t. } out_i > 1$$

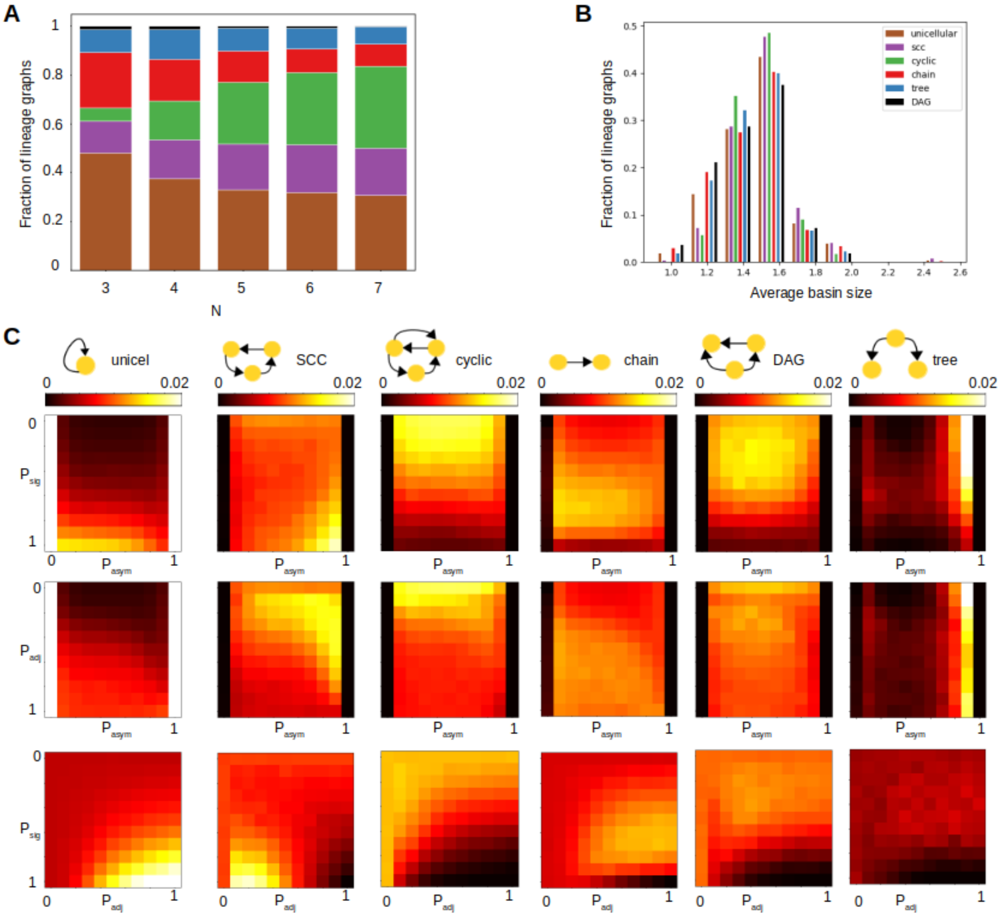

Figure S6: **Lineage graph topologies**. 4852994 graphs were used to generate these plots. **(A)** Stacked histogram for topologies of lineage graphs obtained with different N. Different topologies are represented with different colours: unicellular:brown, SCC:purple, cyclic:green, chain:red, DAG:blue, tree:black. Heights of colored blocks represent the proportions of corresponding topologies. **(B)** histogram of average basin sizes in the gene regulatory networks of homeostatic organisms with lineage graphs of different topologies. **(C)** 2-D histograms indicating distribution of topologies across parameter space. The first row of histograms show distributions along P_{asym} and P_{sig}, second row along P_{asym} and P_{adj}, and the third row along P_{adj} and P_{sig}. Different columns correspond to histograms for different topologies, as indicated at the top of each column. Intensity of colours in histograms in any column indicates the fraction of graphs of a particular topology found in the corresponding parameter region, according to the colorbars given at the top of each column.

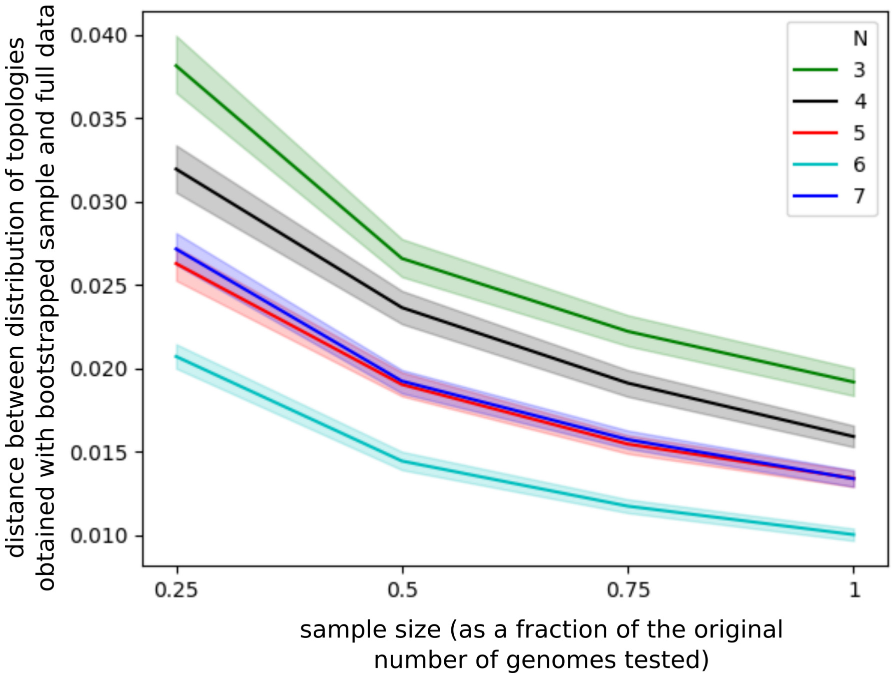

Figure S7: **Effect of genome regulation.** 4852994 graphs were used to generate this plot. Euclidean distance between normalized topology distributions obtained with bootstrap samples and those obtained with the full data for each N. We tested samples with 25%, 50%, 75% and 100% of all GR matrices used in the data for a given N. 1000 samples were generated for each sample size. Lines indicate mean distance and shaded region indicate 95% confidence intervals.

We define the degree of divergence of this graph as $(g_d - g_c)/n_e$. For a perfectly divergent tree, such as the tree to the left in Fig.S10(A), the degree of divergence is 1. And for a perfectly convergent tree (e.g. the tree to the right in Fig.S10(A)), degree of convergence is -1. We find that most tree-like graphs in our data tend to be more convergent than divergent (Fig.S10(B)). Lineage graphs that are divergent lead to an increase in cell-type diversity starting from a few initial cell-types. Lineage graphs of real organisms are believed to be divergent trees. Larger trees tend to be more divergent (Fig.S10(C)). Degree of divergence decreases as P_{asym} increases, it is relatively insensitive to P_{sig} and P_{adj}.

1.7 Characteristics of DAG-type lineage graphs

DAG-type graphs differ from tree-like graphs in having edges that link different branches. If the edges in the DAG are rendered undirected, these edges are parts of cycles, or loops (Fig.S11(A)). The number of such edges in DAGs can be determined by subtracting the number of edges in the spanning tree of the graph from the total number of edges. For a graph with n nodes, the spanning tree has $n - 1$ edges. For a given DAG-type graph, we call the fraction of its edges that forms loops, its link-fraction. Link-fractions of DAG-type lineage graphs indicate the level of trans-differentiation. DAG-type graphs in our data have high link-fractions (Fig.S11(B)), and link-fraction increases with graph size (Fig.S11(C)).

1.8 Distribution of regenerative capacities

. In order to infer whether a lineage graph is regenerative, we only look at whether its regenerative capacity is greater than 1, or not. In Fig.S13(A), we show the spread of regenerative capacities for different topologies. For most topologies, median regenerative capacity is greater than 1. The actual value of regenerative capacity is less meaningful, except in the case of tree-type graphs, where most trees have a regenerative capacity of 0. This implies that most trees contain no pluripotent cells. We also find that while median regenerative capacity decreases with N, the range of regenerative capacities increases with N (Fig.S13(B)).

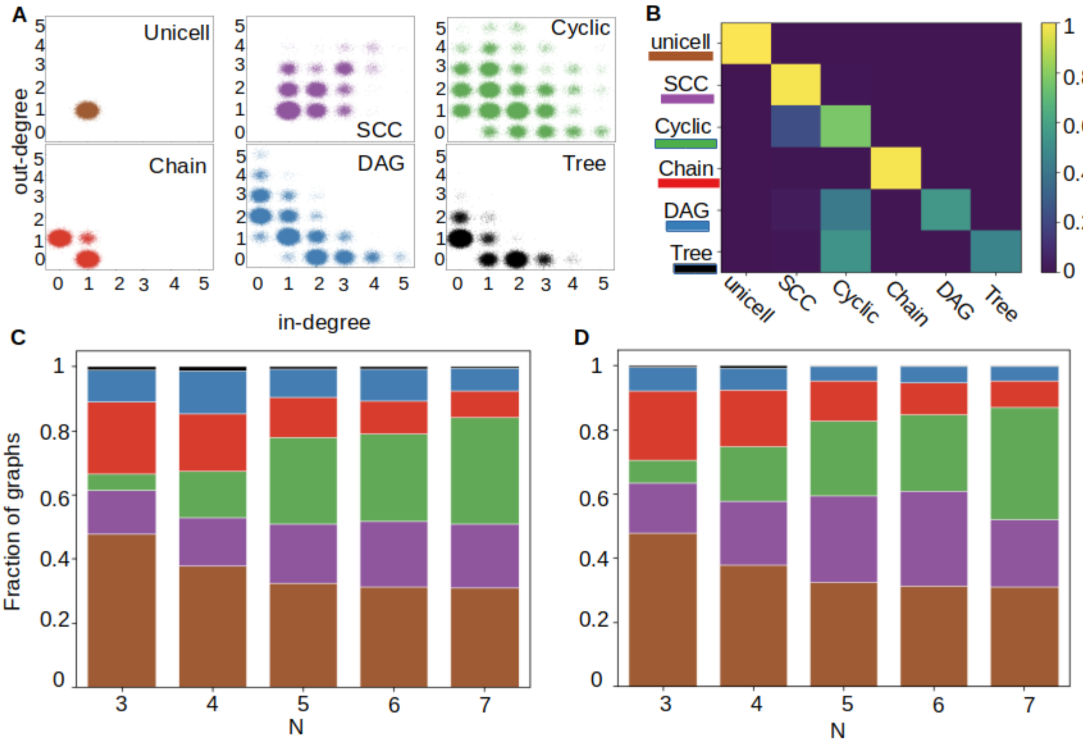

Figure S8: **Distribution of topologies of randomized graphs.** **(A)** Scatter plots for in-degrees and out-degrees of graph nodes in different topologies. Different topologies are represented by different colours: unicellular: brown, SCC: purple, cyclic: green, chain: red, DAG: blue and tree: black. Noise has been added to points in the plots to make the density of points at each position more apparent. 4852994 graphs were used to generate this figure. **(B)** 2-D histogram representing conversions of graph topology due to randomization. Rows indicate the topologies of original graph and columns indicate the topologies of randomized versions. Intensity of colours in the histogram indicates the fraction of conversions of each type, according to the colorbar given alongside. **(C,D)** Stacked histograms of graph topologies. Heights of coloured blocks indicate the proportion of graphs of the corresponding topology. **(C)** lineage graphs generated by the model, **(D)** randomized lineage graphs. 2373473 graphs were used here.

1.9 Intrinsically independent cell-types are enriched in lineage graphs

We wondered whether the large number of independent cell-types in lineage graphs in our data could be attributed to an insensitivity of these cell-types to signals from other cell-types. Alternatively, these cell-types could be independent despite being responsive to signals from other cell-types. We find that the former case tends to be true. We call a cell-type *intrinsically independent* if the full set of signals that can potentially be received by each of its daughter cells is already satisfied by signaling among these daughter cells themselves. In other words, no further external signals can influence the fates of the daughter cells of intrinsically independent cell-types. We calculated the fraction of intrinsically independent cell types across all 2^N possible cell-types across all systems in our data. We find that cell-types that are part of lineage graphs are much more likely to be intrinsically independent irrespective of parameter region (Fig.S14). Thus cell-types in lineage graphs are predisposed to be independent. But, not all independent cell-types in lineage graphs are intrinsically independent (overall, about 20% the independent cells across all lineage graphs are not intrinsically independent), and not all independent cell-types are pluripotent (Fig.S14(A)).

In lineage graphs, most pluripotent cells are independent of cellular context (Fig.S15(B)). Interestingly, while the proportion of independent cell-types pooled from all graphs is similar ($\geq 75\%$) across all topologies, different topologies have very different proportions of pluripotent cells (Fig.S15(A), fourth panel). Notably, in SCC-type lineage graphs, where all differentiation paths are cyclic, 99.8% of all independent cells are pluripotent. Whereas in lineage graphs that contain acyclic differentiation paths, the

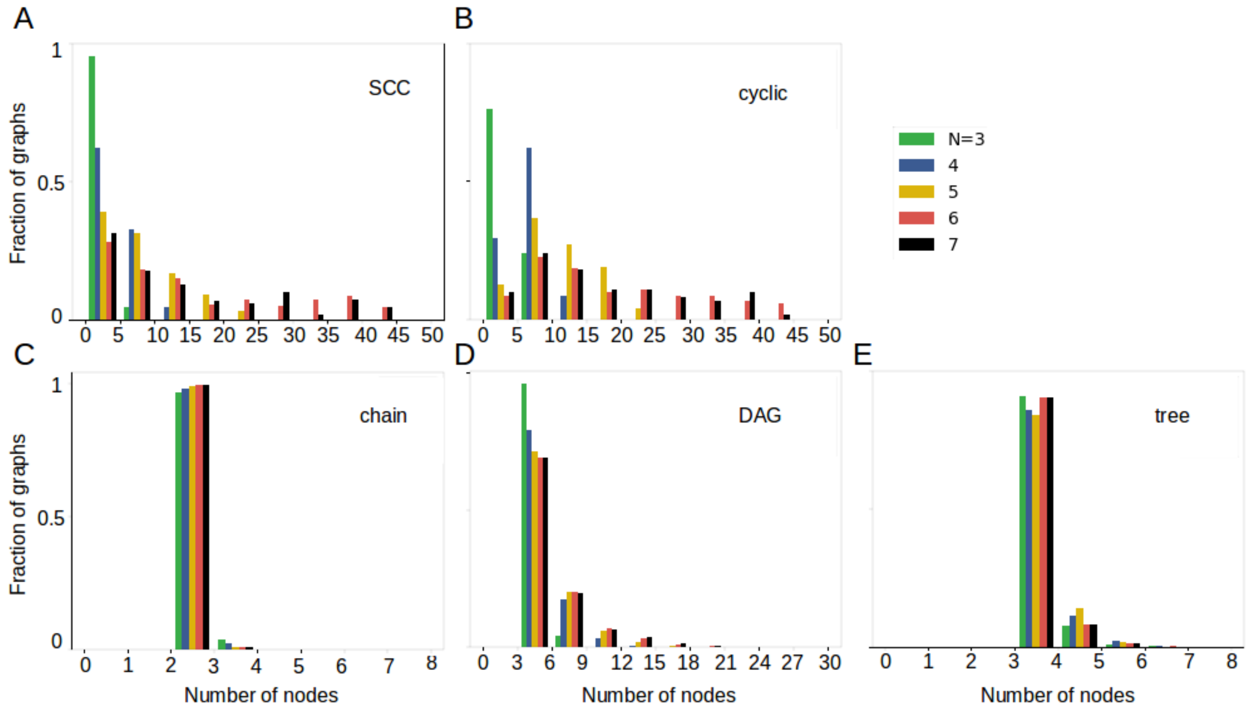

Figure S9: **Graph size distributions for different topologies.** The horizontal axis indicates number of nodes in lineage graphs and the vertical axis indicates the normalized frequency of graphs. Histogram bin sizes are as follows: **(A,B)** 5, **(C)** 1, **(D)** 3, **(E)** 1. Number of graphs used in these figures are: **(A)** 850101, **(B)** 1044178, **(C)** 695972, **(D)** 474582, **(E)** 46554.

proportion of pluripotent independent cells is lower; particularly in tree-type lineage graphs, where only 2.4% of the independent cells are pluripotent. More generally, this indicates that not only the number of independent cell-types, but also their connectivity in the lineage graph is an important factor contributing to an organism's regenerative capacity.

1.10 Rules with higher levels of signaling generate lineage graphs with non-root pluripotent cells

We looked at the distribution of parameters $P_{\rm asym}$, $P_{\rm sig}$, $P_{\rm adj}$ of developmental rules encoding lineage graphs with only pluripotent root nodes, and those with non-root pluripotent nodes. We see no significant difference in the distribution of $P_{\rm asym}$, that generate the two kinds of lineage graphs (Fig.S16(A,D)). But, consistently with our arguments, we find that lineage graphs with non-root pluripotent cells are more likely to be produced by rules with increased signaling (Fig.S16(B,C,E,F)).

1.11 Cell death may increase the proportion of acyclic lineage graphs

Although cells do not undergo programmed death in the current version of the model, here we demonstrate one possible way of implementing programmed cell death, and its implications for lineage graph topologies. We consider 'cell-death' as an additional stable cell-state, and a randomly chosen set of cell-types are assigned to its basin. We implement cell-death concomitantly with the gene regulation step in the current version of the model. Cell-types that map to 'cell-death' are removed from the organism and not carried forward to the updated state of the organism. We find that allowing some cell-types to die, even though we start from the same initial condition, and use identical rules for signaling and cell-division, can result in completely different homeostatic organisms. Importantly, the nodes of these lineage maps are no longer bound to have out-edges (Fig. S17). Such nodes should increase the propensity of the model to produce acyclic graphs.

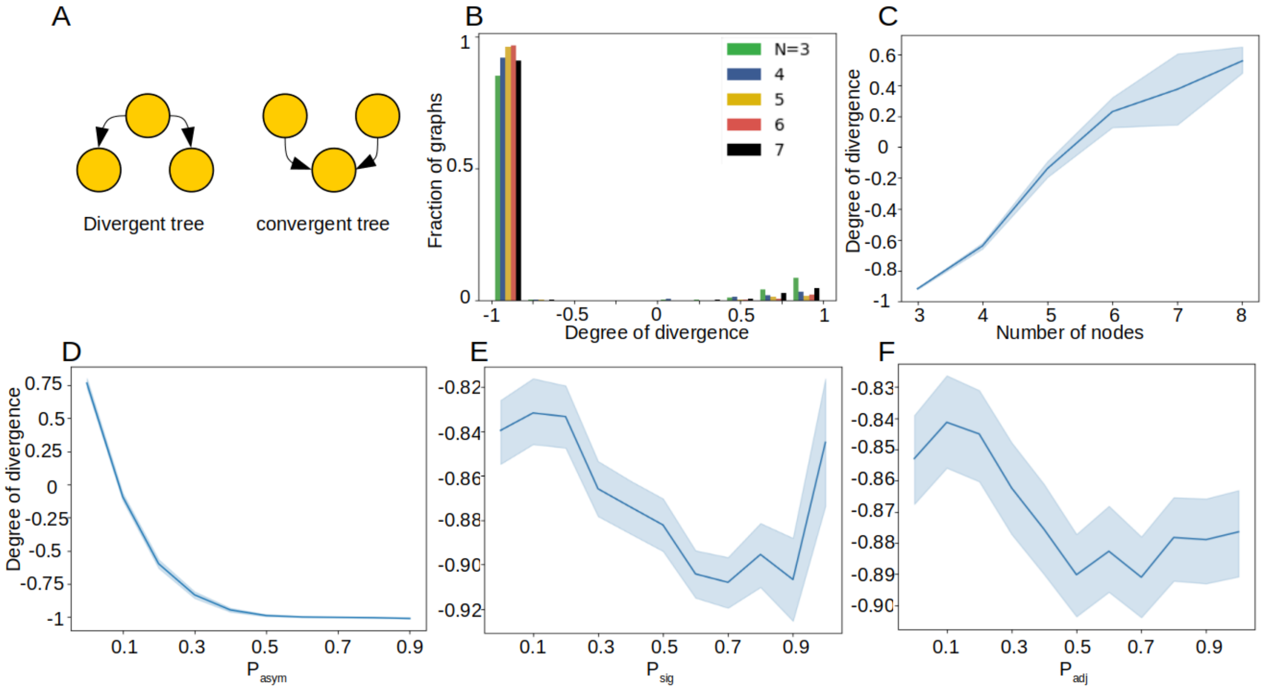

Figure S10: **Properties of tree-type graphs.** 46554 graphs were used to generate these plots. **(A)** Schematics of a divergent tree like lineage graph and a convergent tree like lineage graph. Yellow circles represent cell-types and edges represent lineage relationships. **(B)** Histogram of degrees of divergence for tree-like graphs in our data with different N. **(C,D,E,F)** Average degree of divergence in our data as a function of **(C)** number of nodes in lineage graphs, **(D)** P_{asym}, **(E)** P_{sig}, **(F)** P_{adj}. Shaded regions indicate standard deviation.

1.12 ’Acyclized’ versions of cyclic graphs

We derived ’acyclized’ versions of cyclic graphs in our data by merging all nodes belonging to any SCC (strongly connected component) into a single node. In the acyclized graph, there is an edge from some node A to some node B, if in the original graph there is at least one node in SCC A that gives an edge to at least one node in SCC B. Among these acyclized graphs, 45% are unicellular, i.e. the original graphs had a single SCC. DAGs are the next most abundant acyclized graphs, and trees are the least abundant (Fig. S18A). In order to measure *how cyclic* any graph is, we use two metrics: *acyclic edge fraction*, and *acyclic node fraction* which are the ratios of (number of edges/nodes in the acyclized graph) / (number of edges/nodes in the original graph). For the large fraction of acyclized graphs that are unicellular, evidently, both measures are 0. For non-unicellular acyclized graphs, the acyclic edge fractions tend to be very small (on average 0.29), while acyclic node fraction is on average 0.5 (Fig. S18B). In Fig. S18C, we calculate the fraction of trees across all graphs in the data using relaxed definitions for tree-type graphs. We find that even after substantially relaxing constraints and counting all cyclic graphs with acyclic edge fraction ≥ 0.5 as acyclic graphs, and counting all DAGs with $n_l/n_b \leq 0.5$ as trees, the fraction of trees across all graphs remains very low. We also check whether relaxing the definition of trees effects our results on the regenerative capacities of different topologies (Fig.S19). Although the median fraction of regenerative tree-type graphs increases from 0.1232 to 0.1911, qualitatively, this fraction is still the lowest among all topologies considered.

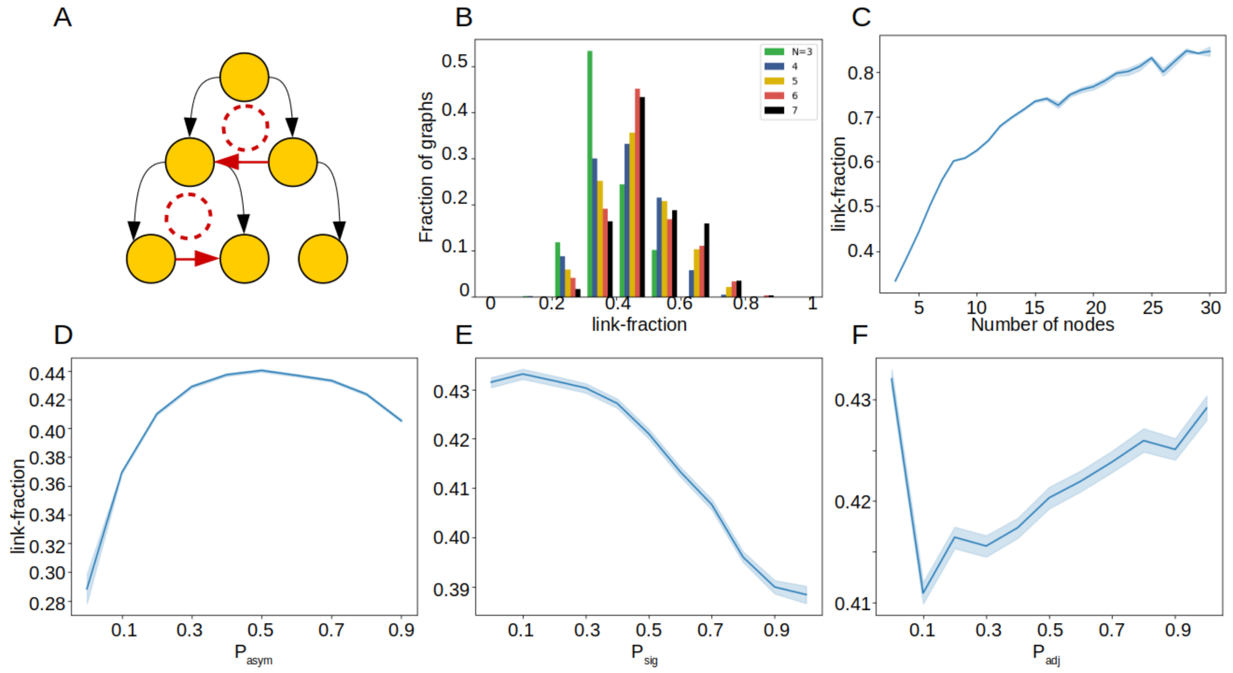

Figure S11: **Properties of DAG-type graphs.** 474582 graphs were used to generate these plots. **(A)** Schematic of DAGs. Yellow circles represent cell-types, and edges represent lineage relationships. Red edges forms loops in the DAG. **(B)** Histogram of link-fraction of DAGs in our data. Loop-fraction is defined as the fraction of edges in a DAG that form loops. Histogram bins are of size 0.1. **(C,D,E,F)** Average loop-fraction of DAG type graphs in our data as a function of **(C)** number of nodes in lineage graphs, **(D)** P_{asym}, **(E)** P_{sig}, **(F)** P_{adj}. Shaded regions indicate standard deviation.

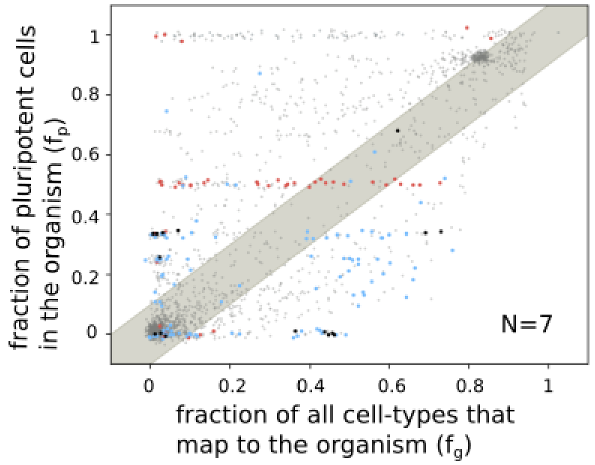

Figure S12: **Regenerative capacity and isomorphic graphs**. 13177 graphs were used to generate this plot. Here, we plot the same graph as in Fig3(A) in the main paper, but only include points for graphs that are not isomorphic to any other graph with the same values of f_g and f_p. This reduces the density of points at some of the 'clusters' which appear in Fig4(A).

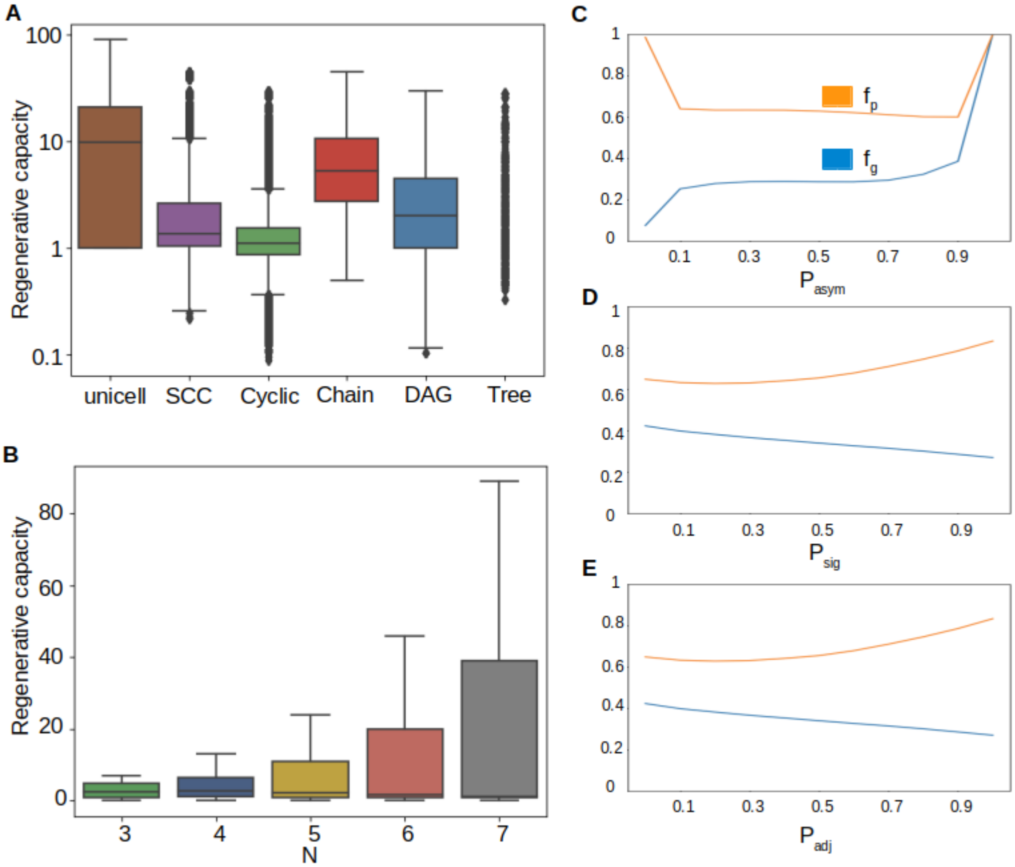

Figure S13: **Box plots for regenerative capacity of lineage graphs.** 4852994 graphs were used to generate these plots. **(A)** across different topologies, **(B)** across number of genes N. Boxes represent quartiles of the data set. Lines inside the box shows the median, while whiskers show the rest of the distribution. Outliers are shown as diamonds. Most tree-like lineage graphs have a regenerative capacity of 0, therefore the box for these graphs is not visible.**(C,D,E)** Variation of regenerative capacity across model parameters: **(C)** P_{asym}, **(D)** P_{sig}, **(E)** P_{adj}. Fraction of pluripotent cells (f_p) is shown in orange, and the fraction of all cells (present or absent from organisms) that develop into the organism (f_g) is shown in blue. Bold lines represent mean values (shaded regions around the lines represent standard deviations, which are small and hardly noticeable). The average regenerative capacities of graphs at different parameter values can be judged by the difference in the heights between the orange and blue curves.

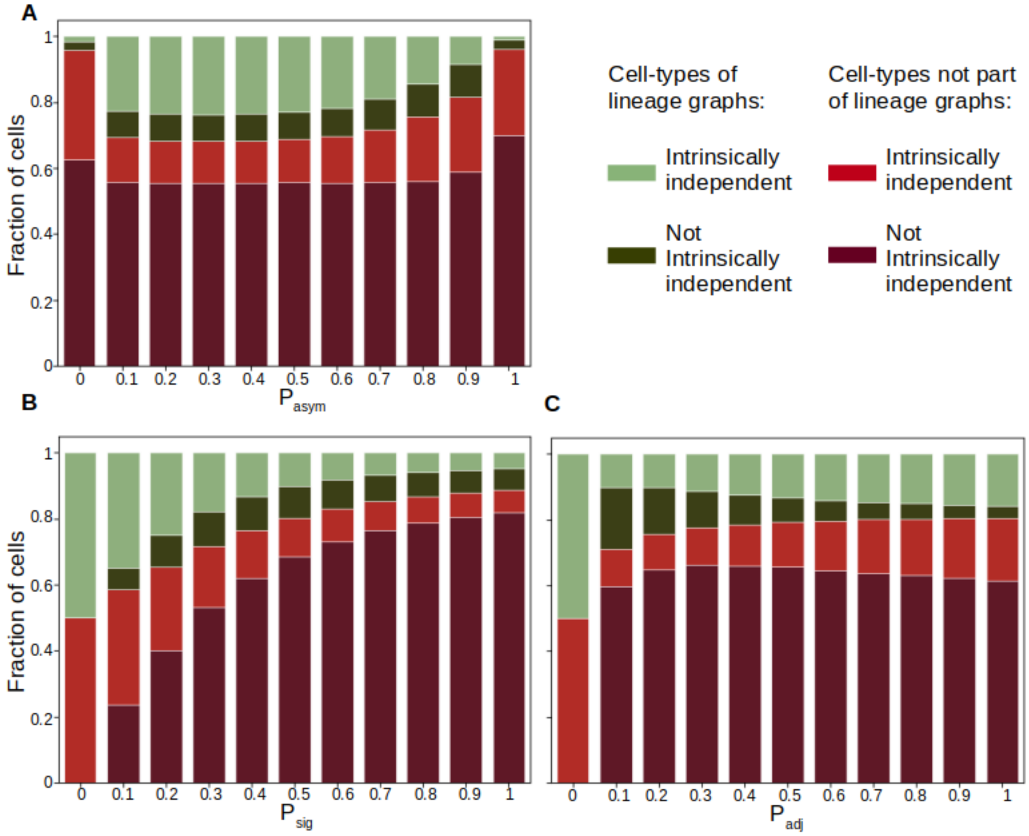

Figure S14: **Stacked histograms showing intrinsic independence of cell-types**. 4852994 graphs were used to generate these plots. **(A)** as a function of P_{asym}, **(B)** as a function of P_{sig}, **(C)** as a function of P_{adj}. Different cell-type categories are represented with different colours. Cell-types not part of organisms are represented in reds; intrinsically independent: bright red, not intrinsically independent: dark red. Cell-types found in organisms are represented in greens; intrinsically independent: light green, not intrinsically independent: dark green. Heights of colored blocks represent the proportions of corresponding cell-types.

References

Réka Albert and Hans G Othmer. The topology of the regulatory interactions predicts the expression pattern of the segment polarity genes in drosophila melanogaster. *Journal of theoretical biology*, 223(1):1–18, 2003.

P. Erdös and A. Rényi. On random graphs i. *Publicationes Mathematicae Debrecen*, 6:290, 1959.

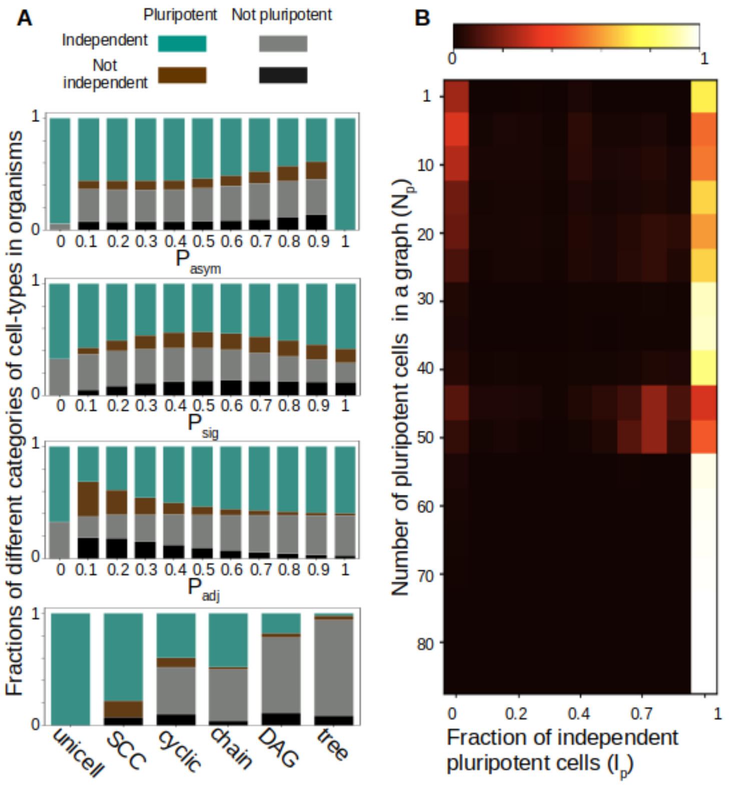

Figure S15: **Independent pluripotent cell-types** 4852994 graphs were used to generate these plots. **(A)** Stacked histograms for cell-types of different categories pooled from organisms across different parameter values (top 3), or across lineage graphs with different topologies (see also Fig.S1, Fig.S2). Different cell-type categories are represented with different colours. Non-pluripotent cells are represented in greys; independent: light grey, not independent: black. Pluripotent cells are represented in colours; independent: teal, not independent: brown. Heights of colored blocks represent the proportions of corresponding cell-types.**(B)** 2-D histogram indicating the fraction of independent pluripotent cell-types in homeostatic organisms. Intensity of colours in the histogram indicate the fraction of organisms with N_p pluripotent cell-types, I_p of which are independent, according to the colorbar given on top.

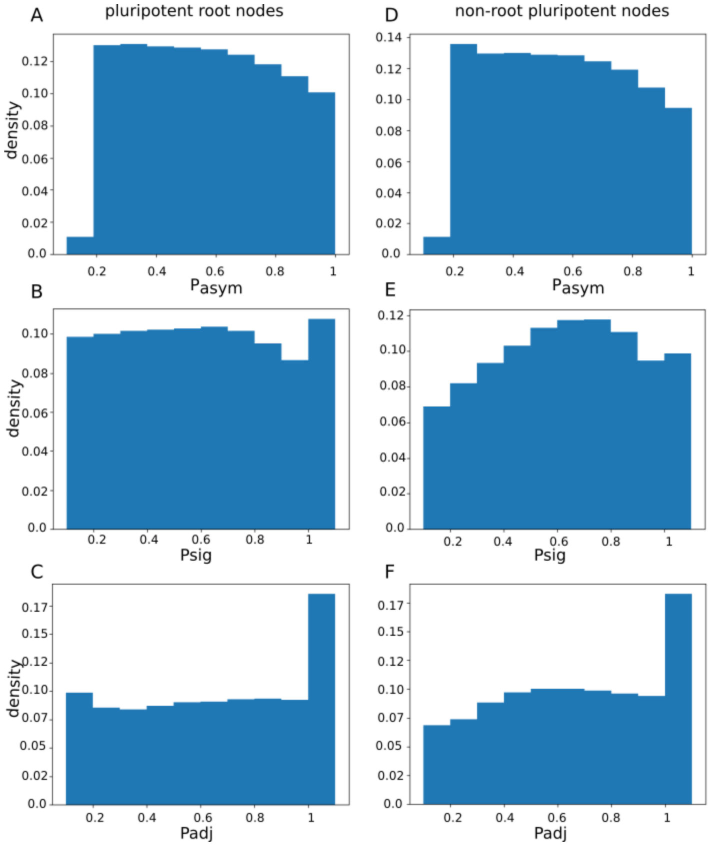

Figure S16: **Comparison of parameters that generate regenerative acyclic lineage graphs with pluripotent root nodes versus those with non-root node pluripotent cells.** **(A,B,C)** Parameter distributions for graphs with pluripotent root nodes. 663849 graphs were used to generate these plots. **(D,E,F)** Parameter distributions for graphs with non-root pluripotent nodes. 463054 graphs were used to generate these plots.

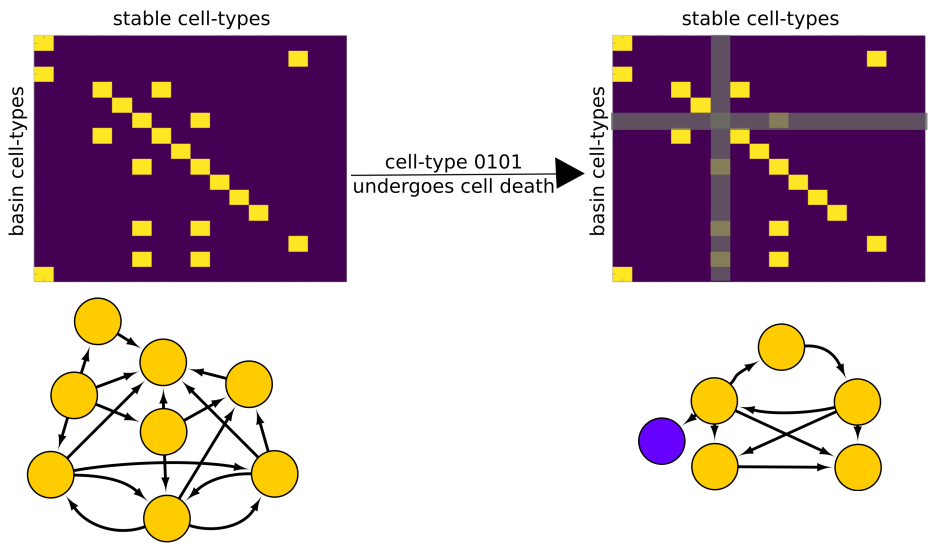

Figure S17: **Effect of including cell-death in the model.** The matrix on the left represents the GR matrix for generating the 'cyclic' graph shown in Fig2(B) in the main paper, and the matrix on the right represents the modified GR, where cell-type 0101 undergoes cell-death. Below the matrices are the lineage graphs generated using the corresponding GR matrices, while the initial condition, and the rest of the rules matrices are kept the same as those used to generate the original 'cyclic' lineage map. In the lineage graph on the right, the purple node has no out-edges (not even a self-edge).

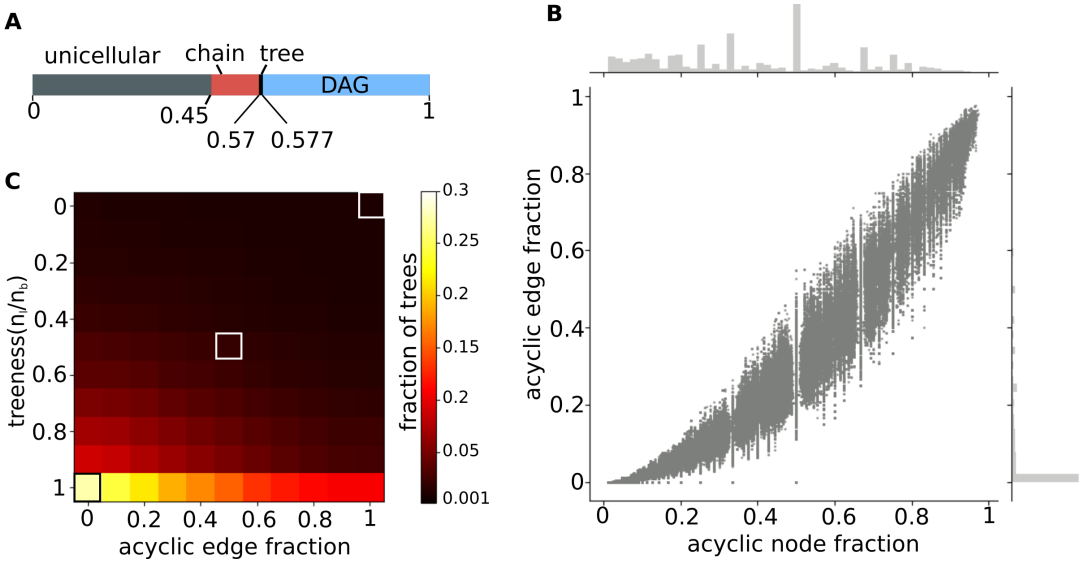

Figure S18: **Properties of 'acyclized' cyclic graphs.** 1894279 graphs were used to generate **(A,B)**. **(A)** Topology distribution of 'acyclized' lineage graphs. **(B)** Scatter plot of *acyclic node fraction* versus *acyclic edge fraction* for non-unicellular acyclized lineage graphs. The histograms opposite the axes represent the marginal distributions for the corresponding axes. **(C)** Heat-map for the fraction of trees across all graphs in the data when the definitions of acyclic graphs and tree-type graph are relaxed. The *acyclic edge fraction* threshold for considering a cyclic graph as acyclic relaxes from right to left on the x-axis. The n_l/n_b threshold for considering a branched acyclic graph as a tree relaxes from top to bottom. The white-edged square on the top-right represents the case where strict definitions for acyclic and tree-type graphs are used (fraction of trees is 0.01). In the white edged square in the middle (edge-fraction threshold = 0.5, n_l/n_b threshold = 0.5), the fraction of tree-type graphs is 0.02. The black-edged square on the bottom-left represents the most relaxed case where all cyclic graphs are considered acyclic and all branched acyclic graphs are considered trees. Here, the fraction of tree-type graphs 0.3. 4852994 graphs were used to generate this plot.

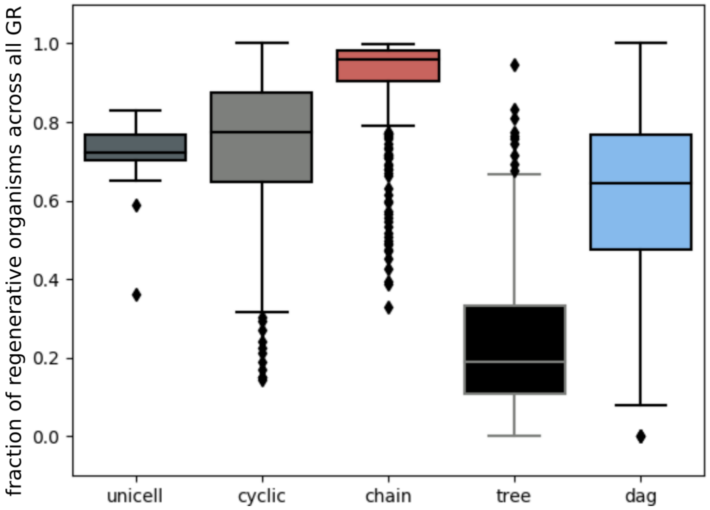

Figure S19: **Box plots for regenerative capacities using relaxed definitions for acyclic graphs and trees.** Here, we treated acyclized graphs with edge-fractions$\geq$ 0.5 as acyclic graphs. In addition, we treated acyclic graphs with $n_l/n_b \leq 0.5$ as trees. For each GR used in our data, for a given graph topology, we looked at the fraction of graphs with regenerative capacity > 1 (as described in the main paper). Boxes represent quartiles of the data set. Lines inside the box show the median, while whiskers show the rest of the distribution. Outliers are shown as diamonds. 4852994 graphs were used to generate this plot.
